# Retinal Microvascular Changes in Mild Cognitive Impairment and Alzheimer's Disease: A Systematic Review, Meta-Analysis, and Meta-Regression

**DOI:** 10.3389/fnagi.2022.860759

**Published:** 2022-04-28

**Authors:** Tsai-Chu Yeh, Chun-Tung Kuo, Yu-Bai Chou

**Affiliations:** ^1^Department of Ophthalmology, Taipei Veterans General Hospital, Taipei, Taiwan; ^2^School of Medicine, National Yang Ming Chiao Tung University, Taipei, Taiwan; ^3^Institute of Health Behaviors and Community Sciences, College of Public Health, National Taiwan University, Taipei, Taiwan; ^4^School of Public Health, National Defense Medical Center, Taipei, Taiwan

**Keywords:** Alzheimer's disease, mild cognitive impairment, retinal microvasculature, retina, optical coherence tomography angiography (OCTA)

## Abstract

**Background:**

The remarkable increase in prevalence and significant morbidity of neurodegenerative diseases pose a tremendous burden for the health care system. Changes in retinal microvasculature metrics associated with Alzheimer's disease (AD) and mild cognitive impairment (MCI) may provide opportunities for early diagnosis and intervention. However, the role of retinal vascular biomarkers remains controversial. We aim to perform a systematic review, meta-analysis and meta-regression to evaluate the comprehensive retinal microvasculature changes in patients with AD and MCI.

**Methods:**

We conducted a literature search on PubMed, MEDLINE, and EMBASE to identify studies published before May 2021 which assessed the measurements of optical coherence tomography angiography (OCTA) between AD, MCI with healthy control eyes, including foveal avascular zone (FAZ), vessel density (VD) of peripapillary, superficial and deep capillary plexus, and choroidal thickness using a random-effect model. We also performed meta-regression and subgroup analysis and assessed heterogeneity and publication bias to evaluate potential sources of bias.

**Results:**

Compared with control eyes, VD of superficial capillary plexus was significantly lower in AD [standardized mean difference (SMD): −0.48; 95% CI (−0.70 to −0.27); *p* = 0.04] and MCI eyes [SMD: −0.42; 95% CI (−0.81 to −0.03); *p* = 0.03], as well as reduced VD of deep capillary plexus [SMD: −1.19; 95% CI (−2.00 to −0.38]; *p* < 0.001], [SMD: −0.53; 95% CI (−0.85 to −0.22); *p* < 0.001]. FAZ was significantly enlarged in AD eyes [SMD: 0.54; 95% CI (0.09 to 0.99); *p* = 0.02]. The meta-regression analysis showed that the OCTA machine type and macular scan size significantly influenced the variation of VD and FAZ between AD and control eyes (*p* < 0.05).

**Conclusion:**

Our results highlight the potential of OCTA as a biomarker to detect early microvasculature deficits in AD and MCI. Notably, the macular scan size and different OCTA machine type could explain the heterogeneity observed in literatures. This information might be useful for future longitudinal study design to evaluate the role of OCTA in monitoring disease progression and treatment efficacy.

## Highlights

- Compared with control eyes, vessel density of superficial capillary plexus and deep capillary plexus were significantly lower in both Alzheimer's disease (AD) and mild cognitive impairment (MCI) subjects.
- Despite the strong correlation observed, the results of our meta-regression showed that the macular scan area and OCTA machine type had a significant impact on the effect sizes regarding the differences of vessel density (VD) and foveal avascular zone area in AD and MCI subjects.
- Although a lower VD of the deep capillary plexus (DCP) of AD compared to MCI did not reach the significant level, a strong correlation was seen in the forest plot and may likely present significance with more longitudinal studies in the future.

## Introduction

Alzheimer's Disease (AD) is the most common subtype of dementia, clinically characterized by progressive cognitive deficits, with an estimated 24 million individuals affected worldwide (Mayeux and Stern, 2012; Hebert et al., 2013). Mild cognitive impairment (MCI) is considered a transitional stage between normal aging and dementia and is associated with a high risk of progression to AD (Gauthier et al., 2006). Although its pathological changes precede the onset of dementia, early detection remains challenging. Because the AD diagnosis mainly relies on examinations such as cerebrospinal fluid (CSF) and Positron emission tomography (PET) scan, which are expensive and invasive procedures that are not applicable as large-scale screening tools. Currently available therapies for AD aim to initiate early intervention and maximize the remaining activity of the neurons to prevent memory decline (Fish et al., 2019). Hence, it is imperative to discover new biomarkers that can identify individuals with earlier stages of AD who would more likely benefit from potential treatments.

Due to its embryologic origin and cellular resemblances with the brain, the retina has long been viewed as a window to the central nervous system (CNS; London et al., 2013; Madeira et al., 2015; Trost et al., 2016). Changes in retinal microvascular condition may provide information on various neurodegenerative diseases, including AD, and serve as disease-related biomarkers. Moreover, compared to other CNS structures, the retina can be easily visualized using non-invasive retinal imaging tools.

Over the past decade, there have been enormous advances in ophthalmic imaging techniques with enhanced contrast, resolution, and accessibility. Among all the latest technologies, optical coherence tomography angiography (OCTA) has been a landmark discovery in ophthalmology. OCTA enables the characterization of vasculatures in layered retinal structure at the micrometer level within seconds, providing a quantitative and qualitative assessment of the microvascular structure in the retina (Spaide et al., 2018). With these ability, OCTA is a powerful tool to detect neurodegenerative process of AD in the CNS.

Since then, studies have reported abnormalities on OCTA of AD and MCI patients, suggesting the association of retinal microvascular dysfunction and neurodegenerative changes (Bulut et al., 2018; Jiang et al., 2018a; Lahme et al., 2018; Yoon et al., 2019; Zabel et al., 2019; Zhang et al., 2019; Chua et al., 2020; Criscuolo et al., 2020; van de Kreeke et al., 2020; Wu et al., 2020; Shin et al., 2021; Wang et al., 2021). Bulut et al. (2018) were the first to use OCTA to demonstrate retinal vascular changes in AD. They found that decreased vessel density (VD) in superficial capillary plexus (SCP) significantly correlates with Mini-Mental State Examination (MMSE) scores in AD patients, and foveal avascular zone (FAZ) is enlarged compared to healthy controls (Bulut et al., 2018). Although the underlying mechanisms in this association remain unknown, one proposed hypothesis regards the deposition of β-amyloid (Aβ) plaques, which have been found to accumulate in cerebral blood vessels and the retina, mainly in the ganglion cell layer (GCL) and retinal nerve fiber layer (RNFL; Koronyo-Hamaoui et al., 2011; Gupta et al., 2016; Mirzaei et al., 2019). While there is evidence reporting changes in VD and FAZ areas in early AD, studies carried out by some other groups failed to detect a significant difference in these measurements (Querques et al., 2019; Wu et al., 2020). Given the inconsistent results regarding OCTA characteristics in AD and MCI, the role of retinal vascular biomarkers remains inconclusive.

In view of the controversies and limitations in previous studies, we aim to conduct a systematic review and meta-analysis to evaluate retinal microvascular changes on OCTA in AD and MCI patients. In addition, we evaluated the effect of potential confounders, including MMSE score, age, gender, OCT model, and macular scan size using meta-regression.

## Methods

### Eligibility Criteria

We conducted a systemic review and meta-analysis to assess the OCTA measurements in AD and MCI, in accordance with the guidelines presented by the Cochrane Collaboration TC (2011) and Meta-analysis Of Observational Studies in Epidemiology (MOOSE) guideline (Stroup et al., 2000).

We included studies that fulfilled all the inclusion criteria listed below:

1. Original study with case-control design.
2. Primary aim of evaluating the measurements of OCTA in subjects with AD, MCI in addition to controls.
3. AD, MCI subjects diagnosed according to established diagnostic criteria [e.g., Diagnostic and Statistical Manual of Mental Disorders (DSM)-III, DSM-IV, National Institute on Aging and Alzheimer's Association (NIA-AA), National Institute of Neuro- logical, Communicative Disorders and Stroke-Alzheimer Disease and Related Disorders Association (NINDS-ADRDA), Petersen Criteria, The modified Telephone Interview for Cognitive Status (TICS-M), and Clinical Dementia Rating (CDR)].
4. OCTA measurements reported in mean and standard deviation (SD).
5. Studies on human subjects.

The exclusion criteria were as follows:

1. Studies reported only TD-OCT or SD-OCT data.
2. Review articles, case reports, conference abstracts, letters to the editor, and all other study types that did not add original data.
3. Studies that failed to exclude poor-quality OCTA images.
4. Studies diagnosed AD or MCI with unestablished diagnostic criteria.
5. Studies including individuals with cardiovascular disease, cerebrovascular disease or diabetes mellitus were excluded.

### Search Strategy

**Figure 1:**
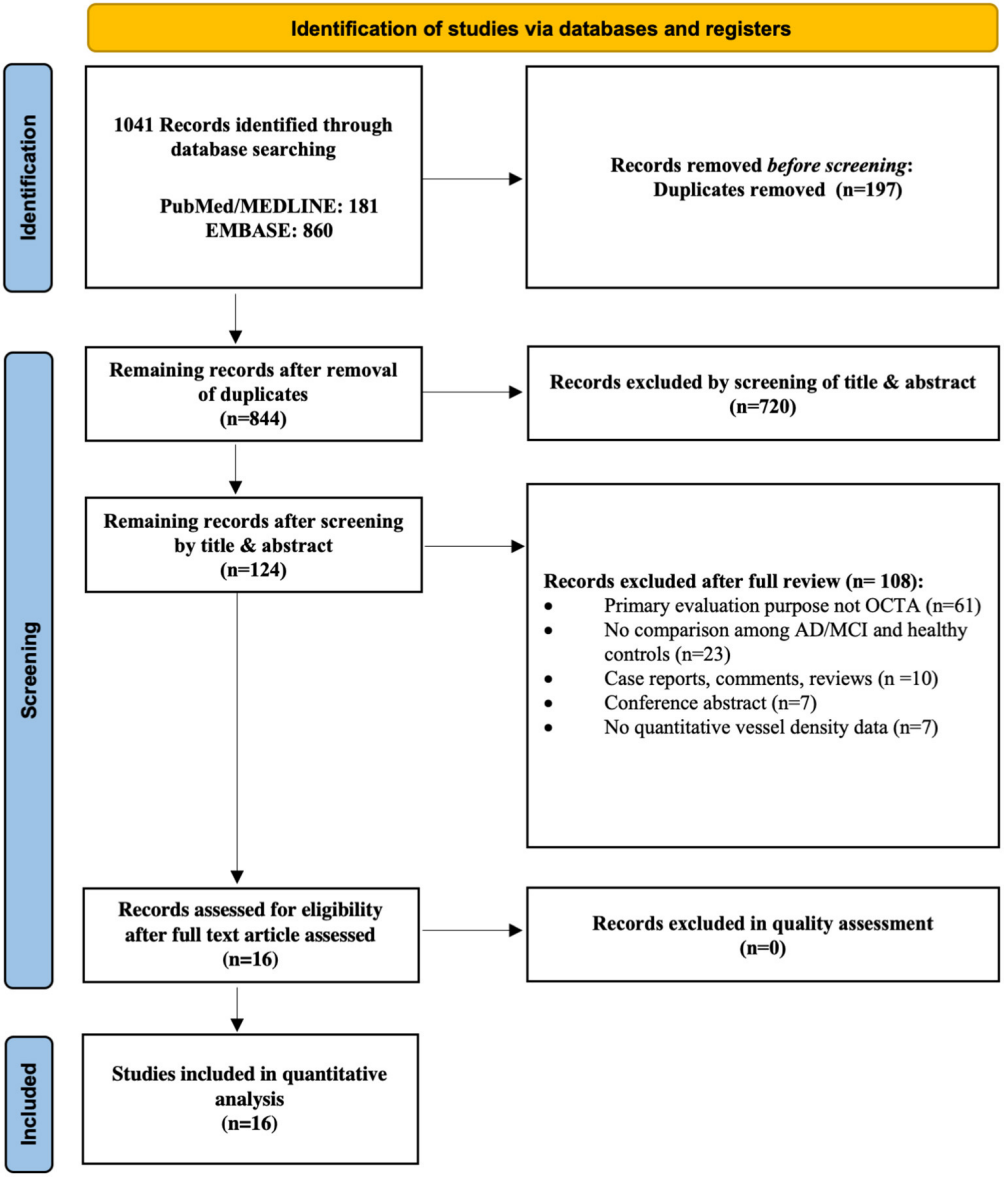
Flow chart of search strategy. The flow chart illustrates the search strategy according to the guidelines of the Preferred Reporting Items for Systematic reviews and Meta-Analyses (PRISMA) 2020 statement.

Two independent reviewers (TCY and CTK) conducted a systematic and comprehensive search in PubMed and EMBASE database from 1 January 2014 to 1 May 2021. We used the medical subject headings (MeSH) for the PubMed query with the following (Tomography, Optical Coherence [MeSH] AND (Retinal Diseases[MeSH Terms]) OR (Retinal Vessels[MeSH Terms])) AND (((((Dementia[MeSH Terms]) OR (Alzheimer's Disease[MeSH Terms])) OR (Cognitive Dysfunction[MeSH Terms])) OR (“Cognitive Impairment”)) OR (“Cognitive Decline”)). The literature search was limited to English-written full-text manuscripts that were published in peer-reviewed journals. The list of the detailed search strategy is presented in Figure 1 in accordance with the Preferred Reporting Items for Systematic Reviews and Meta- Analysis (PRISMA). To reduce the chance of missing relevant articles, we also manually searched the reference lists of all gray literature, including relevant articles and reviews. No ethical approval was required for this review study.

### Data Extraction

Two reviewers (TCY and CTK) independently read each article to ascertain its eligibility and extracted data. Disagreements were resolved by discussions with a senior reviewer (CYB). Extracted information included authors, the title of the study, publication year, country, method of eye selection (i.e., analysis including one eye or both eyes of each subject), number of subjects in each population, mean age, gender ratio, mean MMSE score, OCT model, macular scan size (3x3 or 6x6 mm^2^), method of VD assessment (whether provided by the device or calculated by software or manually), and minimum SSI accepted for a quality scan. The Newcastle-Ottawa Scale criteria were used to assess the quality of the included case-control studies, with a scale range of 0–9 points (Table 1; Stang, 2010; Wells et al., 2016).

**Table 1:** Newcastle-Ottawa Quality Assessment Scale of case control studies.

**Study**	**Selection**				**Comparability**	**Outcome**			**Quality**
	**1**	**2**	**3**	**4**	**1**	**1**	**2**	**3**	**Score**
Bulut et al. (2018)	1	1	0	1	2	1	1	0	7
Lahme et al. (2018)	1	1	0	0	2	1	1	0	6
O'Bryhim et al. (2018)	1	1	0	1	2	1	1	0	7
Jiang et al. (2018b)	1	1	1	1	2	1	1	0	8
Querques et al. (2019)	1	1	0	1	2	1	1	0	7
Haan et al. (2019)	1	1	0	1	1	1	1	0	6
Zhang et al. (2019)	1	1	0	1	2	1	1	0	7
Zabel et al. (2019)	1	1	0	1	2	1	1	0	7
Yoon et al. (2019)	1	1	0	1	1	1	1	0	6
Wu et al. (2020)	1	1	0	1	2	1	1	0	7
Chua et al. (2020)	1	1	0	0	2	1	1	0	6
Robbins et al. (2021)	1	1	0	1	1	1	1	0	6
Salobrar-Garcia et al. (2020)	1	1	0	1	1	1	1	0	6
Criscuolo et al. (2020)	1	1	0	1	2	1	1	0	7
van de Kreeke et al. (2020)	1	1	0	0	1	1	1	0	5
Wang et al. (2021)	1	1	0	1	2	1	1	0	7
Shin et al. (2021)	1	1	0	1	2	1	1	0	7
O'Bryhim et al. (2021)	1	1	0	1	2	1	1	0	7

*Criteria for quality assessment:*

*Selection: (1) Is the case definition adequate? (2) Representativeness of the case (3) Selection of controls (4) Definition of Controls*.

*Comparability: (1) Comparability of case controls on the basis of the design or analysis*.

*Outcome: (1) Ascertainment of exposure (2) Same method of ascertainment for cases and controls (3) Non-Response rate*.

The primary outcome variables of OCTA measurements (in mean and SD) include FAZ, VD in SCP, DCP, and peripapillary. The value of each sub-sector of the measured area, if any, was also extracted. We calculated the average values of multiple subsectors using Review Manager Software (version 5.3; Cochrane Collaboration, Oxford, United Kingdom).

### Statistical Analysis

We performed meta-analyses, heterogeneity analysis, subgroup analyses, assessment of publication bias, and meta-regression by Stata software (StataCorp, Texas; version 16.0). Means and standard deviations were used to calculate the standard mean difference (SMD) with respective 95% confidence interval (CI) for continuous variables. We applied random-effect models in all meta-analyses with the restricted maximum likelihood (REML) method. The REML estimation has previously been advocated for continuous outcomes (Veroniki et al., 2016). We tested between-group heterogeneity using the *I*^2^ test. The *I*^2^ statistic ranges from 0 to 100%, with 25, 50, and 75% indicating low, moderate, and high between-study heterogeneity. In addition, we assessed potential publication bias using Egger's Tests.

Random-effects meta-regression was performed to assess the potential confounders on the effect sizes. The included variables were mean MMSE scores, mean age differences between study groups, gender ratio differences, OCT model, and macular scan size. We performed subgroup analyses according to the types of OCTA model and macular scan size. Additionally, we performed sensitivity analyses to account for unmeasured confounding, as random-effects meta-analyses of observational studies can result in biased estimates if the synthesized studies contain unmeasured confounding (Mathur and VanderWeele, 2020). We determined the minimum strength for an unmeasured confounder on the risk ratio scale (*E*-value) required to eliminate the SMDs between case and control groups. The E-value was defined as the minimal magnitude of association that an unmeasured confounder needs to have with both the exposure and the outcome to fully explain a specific exposure-outcome association (Mathur and VanderWeele, 2020). *P*-values < 0.05 were indicated as statistically significant, and all statistical analyses were two-tailed.

### Risk of Bias Assessment

**Figure 2:**
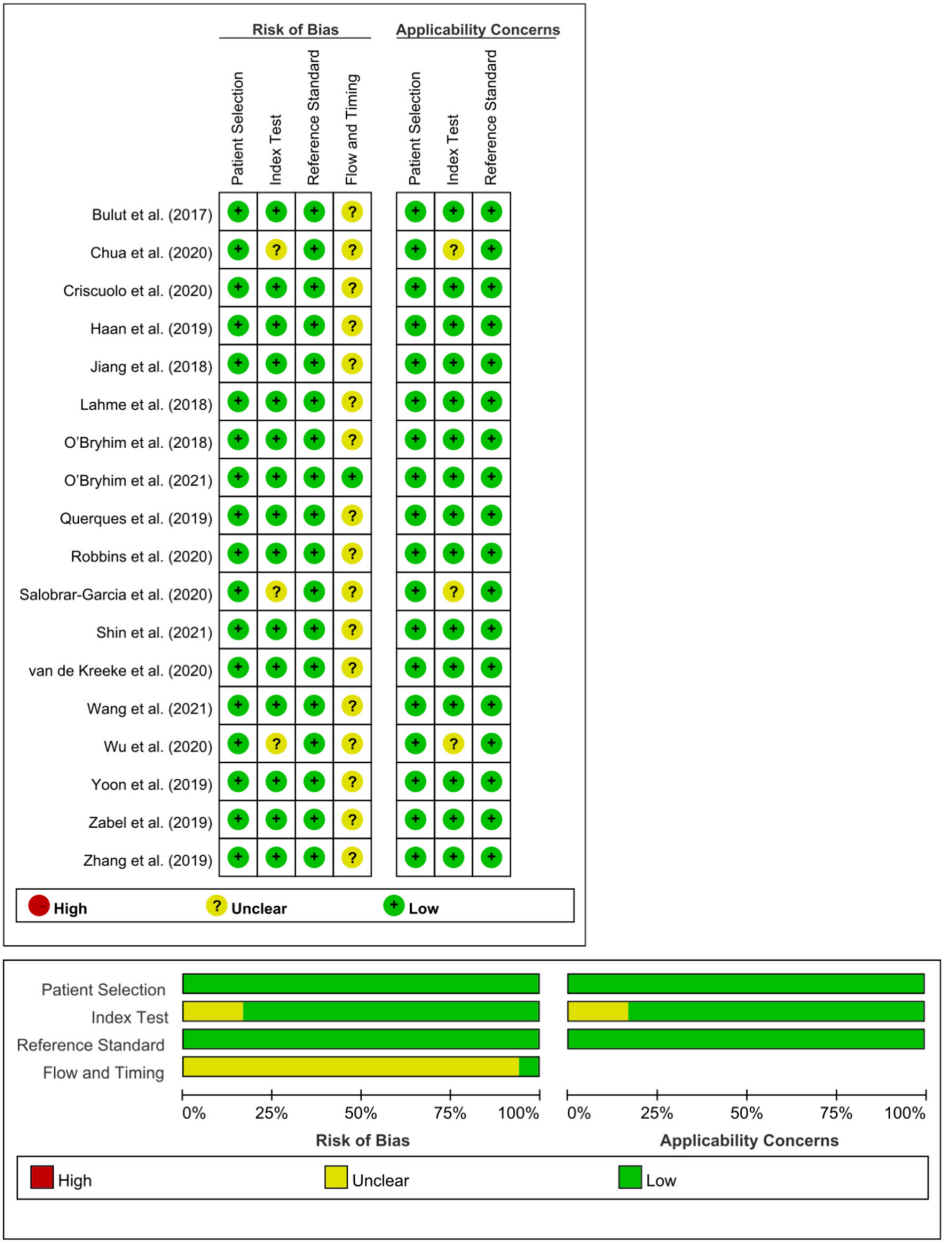
Risk of bias summary using the QUADAS-2 assessment. We assessed study quality using the QUADAS-2, which evaluates risk of bias and applicability issues in patient selection, index test, reference standard, and flow and timing.

A risk of bias assessment for each study was performed by applying a standardized QUADAS-2 assessment (Figure 2). TCY and CTK independently assessed the risk of bias, and disagreements were resolved by discussions with a senior reviewer (CYB). Egger's test was used to evaluate publication bias.

## Results

### Study Selection

Figure 1 illustrates the selection process for the 16 studies included in the meta-analysis. A total of 1041 relevant articles were initially identified across all databases. After removal of duplicates, the remaining 844 studies were screened based on their titles and abstracts. A total of 828 studies were then excluded for various reasons including those not related to AD, MCI, and OCTA, yielding 16 case-control studies involving 444 AD subjects, 391 MCI subjects and 770 controls ultimately eligible for the quantitative analysis. The study by van de Kreeke et al. (2020) was not included in the meta-analysis as it did not report average vessel density between groups. In addition, although Jiang et al. (2018b) measured SCP and DCP in both AD and MCI patients, the original data was not provided to conduct meta-analyses. Despite the possible duplicate populations of authors such as O'Bryhim et al. (2018, 2021), we extracted different outcome variables in these studies. The relevant studies excluded due to insufficient data to perform meta-analyses were still included in the systemic review, and the main characteristics of all studies are presented in Table 2.

**Table 2:** Summary of included case control studies on individuals with Alzheimer's disease and mild cognitive impairment.

**References**	**Country**	**Sample**	**Mean MMSE (±SD)**	**Mean age (±SD)**	**Sex (M/F)**	**Eye**	**Neurocognitive diagnosis**	**OCTA** ** model**	**Macular scan size**	**Parafoveal VD: inner; outer diameter (mm)**	**Choroidal perfusion density**	**SCP**	**DCP**	**FAZ**	**NOS score**
Bulut et al. (2018)	Turkey	26 ADs	16.92 (±7.39)	74.23 (±7.55)	11/15	S	NIA-AA	RTVue XR Avanti	6x6	NA	NA	SCP were significantly lower in AD than controls.	NA	FAZ was significantly enlarged in AD than controls.	7
		26 Controls	26.81 (±2.20)	72.58 (±6.28)	13/13										
Lahme et al. (2018)	Germany	36 ADs	22.32 (±4.45)	67.97 (±9.3)	21/15	S	NIA-AA	RTVue XR Avanti	3x3	NA	NA	SCP were significantly lower in AD than controls.	No significant difference.	No significant difference.	6
		38 Controls	NA	66.08 (±10.11)	23/14										
O'Bryhim et al. (2018)	USA	14 Pre-ADs	NA	73.5 (±4.7)	8/6	P	NA	RTVue XR Avanti	6x6	NA	NA	NA	NA	FAZ was enlarged in biomarker (+) eyes compared with biomarker (-) eyes.	7
		16 Controls	NA	75.4 (±6.6)	6/10										
Jiang et al. (2018b)	USA	12 ADs	NA	73.3	NA	P	NIA-AA	Cirrus 5000 Angioplex	3x3	NA	NA	SCP were significantly lower in AD than controls.	DCP were significantly lower in AD than controls.	NA	8
		19 MCIs	NA	69.6	NA										
		21 Controls	NA	67.6	NA										
Querques et al. (2019)	Italy	12 ADs	20.7 (±3.6)	72.9 (±7.2)	4/8	S	NIA-AA	Cirrus 5000 Angioplex	3x3; 6x6	3;6	No significant difference.	No significant difference.	No significant difference.	No significant difference.	7
		12 MCIs	24.9 (±2.7)	76.3 (±6.9)	5/7										
		32 Controls	NA	71.6 (±5.9)	17/15										
Haan et al. (2019)	Netherlands	48 ADs	23.0 (±3.0)	65.4 (±8.1)	25/23	P	NIA-AA	Cirrus 5000 Angioplex	6x6	1-3; 3-6	NA	No significant difference.	NA	No significant difference.	6
		38 Controls	29.0 (±1.0)	60.6 (±5.0)	24/14										
Zhang et al. (2019)	USA	3 early ADs/13 aMCIs	26.0	73.03 (± 8.24)	3/13	P	NIA-AA	RTVue XR Avanti	3x3	1;3	NA	SCP were significantly lower in early AD/aMCI than controls.	No significant difference.	NA	7
		16 Controls	NA	73.60 (±7.69)	3/13										
Zabel et al. (2019)	Poland	27 ADs	20.55 (±5.46)	74.11 (±5.87)	6/21	S	NIA-AA	RTVue XR Avanti	6x6	1;3	NA	No significant difference.	DCP were significantly lower in AD than controls.	FAZ was significantly enlarged in AD than controls.	7
		27 Controls	28.39 (±1.04)	74.26 (±7.66)	8/19										
Yoon et al. (2019)	USA	39 ADs	20.1 (±5.9)	72.8 (±7.7)	13/26	P	NIA-AA	Cirrus 5000 Angioplex	3x3; 6x6	3;6	NA	SCP were significantly lower in AD than controls and AD vs. MCI but not between MCI and controls.	NA	No significant difference.	6
		37 MCIs	22.6 (±4.7)	72.8 (±7.7)	17/20										
		133 Controls	29.2 (±1.1)	69.2 (±7.8)	36/97										
Wu et al. (2020)	China	18 ADs	19.67 (±2.45)	69.94 (±6.39)	10/9	P	AD (NINCDS-ADRDA); MCI (Petersen criteria)	RTVue XR Avanti	6x6	1-3; 3-6	NA	No significant difference.	DCP were significantly lower in AD and MCI than controls.	FAZ was significantly largest in AD, followed by MCI, and lastly controls.	6
		21 MCIs	24.76 (±1.09)	67.81 (±5.96)	12/9										
		33 Controls	27.14 (±1.20)	68.67 (±5.85)	11/10										
Chua et al. (2020)	Singapore	24 ADs	20.3 (±6.1)	74.9 (±6.0)	7/17	P	AD (DSM-IV); MCI (Petersen criteria)	Cirrus 5000 Angioplex	3x3	1;2.5	NA	SCP were significantly lower in AD and MCI than controls.	DCP were significantly lower in AD than controls.	No significant difference.	6
		37 MCIs	23.9 (±6.3)	77.9 (±6.4)	21/16										
		29 Controls	24.8 (±4.8)	76.7 (±5.3)	16/13										
Robbins et al. (2021)	USA	67 ADs	19.77 (±7.09)	72.76 (±8.07)	22/45	P	NIA-AA	Cirrus 5000 Angioplex	NA	NA	Choroidal vascularity index was significantly less in AD than MCI.	NA	NA	NA	6
		74 MCIs	24.45 (±5.85)	70.04 (±11.53)	33/41										
		137 Controls	28.96 (±2.73)	69.23 (±7.71)	38/99										
Salobrar-Garcia et al. (2020)	Spain	17 MCIs	26.0	NA	NA	S	MMSE/CDR	Heidelberg Spectralis	NA	NA	NA	NA	NA	No significant difference.	5
		15 Controls	30.0	NA	NA										
Criscuolo et al. (2020)	Italy	27 aMCIs	26.51 (±1.8)	73.0 (± 6.0)	12/15	S	NIA-AA	RTVue XR Avanti	6x6	NA	NA	SCP were significantly lower in MCI than controls.	DCP were significantly lower in MCI than controls.	FAZ was significantly enlarged in MCI than controls.	7
		29 Controls	28.03 (±1.3)	73.1 (± 7.0)	14/15										
van de Kreeke et al. (2020)	Netherlands	12 pre -AD Aβ+	29.0^*^	68.6 (±6.3)	58/66	P	TICS-M/CDR	Cirrus 5000 Angioplex	6x6	1-3; 3-6	NA	Aβ+ participants had significantly higher vessel density than Aβ- individuals in all regions.		No significant difference.	5
		111 pre-AD Aβ-													
Wang et al. (2021)	Netherlands	62 ADs	19.92 (±4.54)	71.81 (±7.98)	27/35	P	NIA-AA	RTVue XR Avanti	3x3	1;3	NA	SCP were significantly lower in AD and MCI than controls.	DCP were significantly lower in AD and MCI than controls.	No difference in FAZ among the three groups.	7
		47 MCIs	28.04 (±1.90)	72.73 (±7.75)	18/29										
		49 Controls	28.67 (±1.0)	69.50 (±5.94)	17/32										
Shin et al. (2021)	Korea	40 MCIs	NA	72.8 (±8.6)	25/15	P	NIA-AA	Cirrus 5000 Angioplex	6x6	1-3; 3-6	NA	SCP were significantly lower in MCI than controls.	• No significant difference. • No significant difference.	FAZ was significantly enlarged in MCI than controls.	7
		37 Controls	NA	69.0 (±10.4)	17/20										
O'Bryhim et al. (2021)	USA	16 Pre-ADs	NA	76.29 (±4.66)	NA	P	NA	RTVue XR Avanti	6x6	NA	NA	NA	• NA • NA	FAZ was enlarged in biomarker (+) eyes compared with biomarker (-) eyes.	7
		19 Controls	NA	75.21 (±4.13)	NA										

*NA, Not Available; CC, Case–control study; PS, Prospective study; MMSE, Mini-Mental State Examination; NIA-AA, National Institute on Aging and Alzheimer's Association (NIA-AA); NINCDS-ADRDA, National Institute of Neuro- logical, Communicative Disorders and Stroke-Alzheimer Disease and Related Disorders Association; CDR, Clinical Dementia Rating; TICS-M, The modified Telephone Interview for Cognitive Status; DSM-IV, Diagnostic and Statistical Manual of Mental Disorders, fourth edition; OCTA, Optical Coherence Tomography Angiography; AD, Alzheimer's Disease; MCI, Mild cognitive impairment; aMCI, Amnestic mild cognitive impairment; Eye; S, OCTA measurements of either single eye were selected; P, OCTA measurements of both eyes were considered; SCP, Superficial capillary plexus; DCP, Deep capillary plexus; FAZ, Foveal avascular zone*.

* *Median*.

In all studies, there were no significant differences in mean age between the AD, MCI, and control groups. The mean MMSE scores in the control group ranged from 24.8 to 30.0, whereas scores in the MCI group ranged from 22.6 to 28.0, and scores in the AD group ranged from 16.9 to 23.0. Regarding the OCTA models, nine studies used the RTVue XR Avanti (Optovue, Inc., Fremont, CA, USA; Lahme et al., 2018; O'Bryhim et al., 2018, 2021; Zabel et al., 2019; Zhang et al., 2019; Criscuolo et al., 2020; Wu et al., 2020; Wang et al., 2021), six studies used the Zeiss Cirrus HD-5000 with AngioPlex (Carl Zeiss Meditec, Dublin, CA; Haan et al., 2019; Querques et al., 2019; Yoon et al., 2019; Chua et al., 2020; Robbins et al., 2021; Shin et al., 2021), and one study used Heidelberg Spectralis (Salobrar-Garcia et al., 2020).

### Superficial Capillary Plexus VD

**Figure 3:**
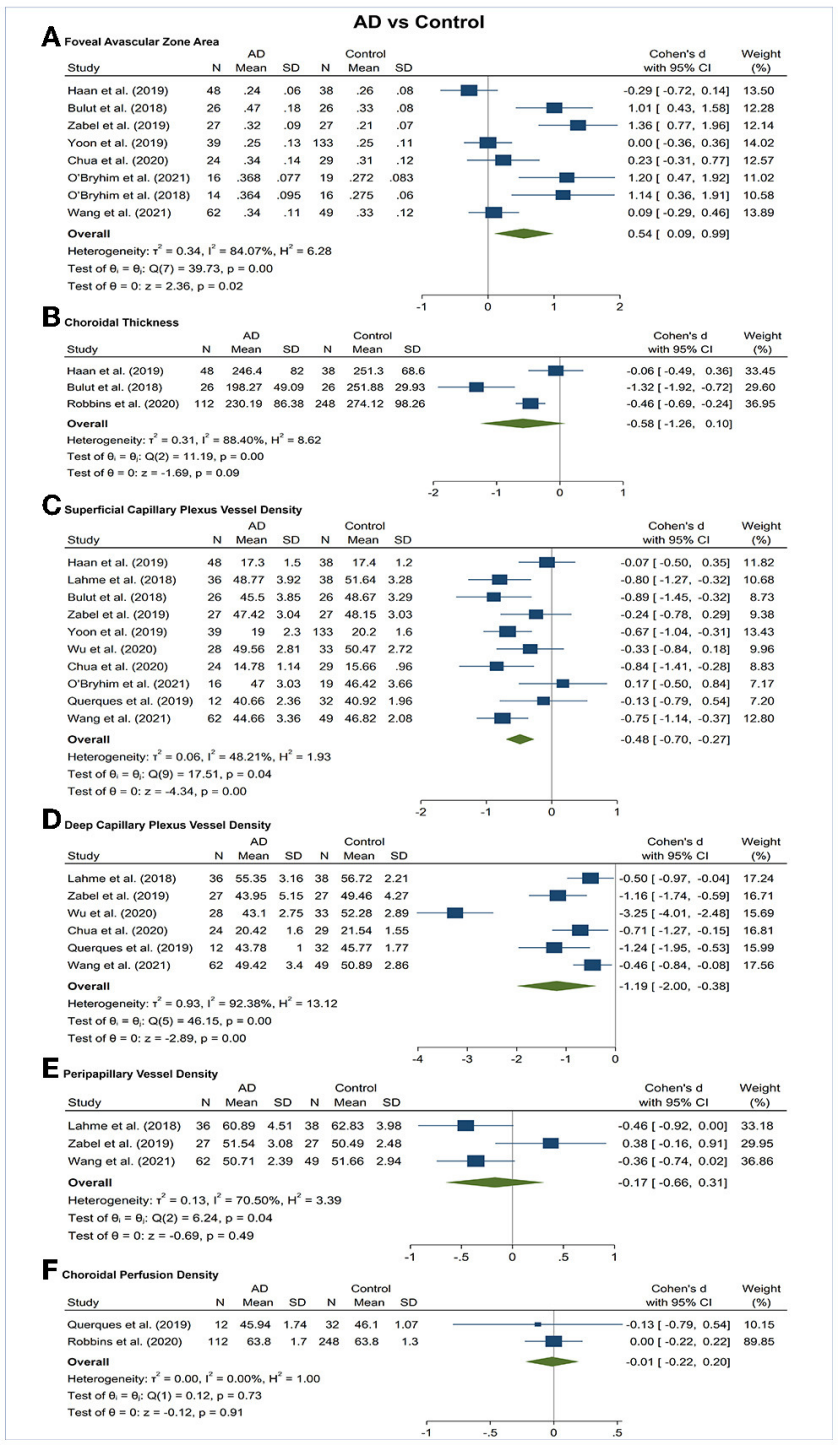
Forest plot of OCTA measurements between subjects with Alzheimer's Disease (AD) and healthy controls. The meta-analyses were conducted with a random-effects model. Horizontal bar indicates 95% confidence intervals (CI), and the size of the squares denotes the weight attributed to each article. The diamonds represent the standardized mean differences with the width showing the 95% CI. **(A)** Foveal avascular zone area. **(B)** Choroidal thickness. **(C)** Superficial capillary plexus vessel density. **(D)** Deep capillary plexus vessel density. **(E)** Peripapillary vessel density. **(F)** Choroidal perfusion density.

**Figure 4:**
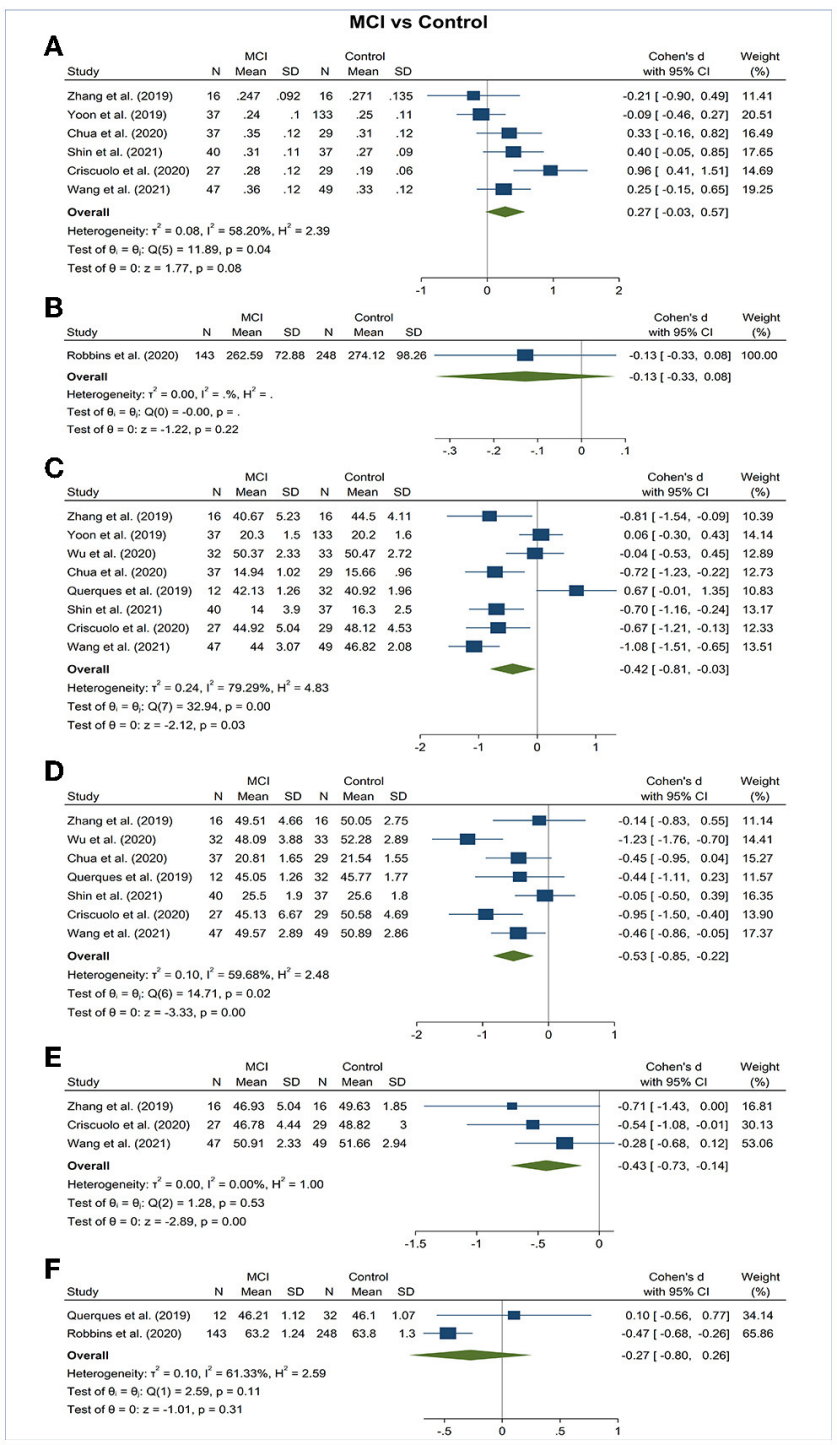
Forest plot of OCTA measurements between subjects with mild cognitive impairment (MCI) and healthy controls. The meta-analyses were conducted with a random-effects model. Horizontal bar indicates 95% confidence intervals (CI), and the size of the squares denotes the weight attributed to each article. The diamonds represent the standardized mean differences with the width showing the 95% CI. **(A)** Foveal avascular zone area. **(B)** Choroidal thickness. **(C)** Superficial capillary plexus vessel density. **(D)** Deep capillary plexus vessel density. **(E)** Peripapillary vessel density. **(F)** Choroidal perfusion density.

Vessel density of SCP significantly lower in the AD and MCI groups compared to controls (Figures 3, 4). Microvascular densities of SCP were significantly lower in AD vs. controls (SMD, −0.48; 95% CI, −0.70 to −0.27; *p* = 0.04; *I*^2^ = 48.21%), and MCI vs. controls (SMD, −0.42; 95% CI, −0.81 to −0.03; *p* = 0.03; *I*^2^ = 79.29%). To nullify the above pooled SMD estimates of vessel density in SCP, an unmeasured confounder would need to be associated with a risk ratio of 2.47 and 2.29 for AD or MCI risk and OCTA retinal measurements, respectively.

The results of subsector and quadrantal analyses revealed a significantly superficial microvascular loss between AD and healthy controls in the fovea (<1 mm from the fovea; SMD, −0.38; 95% CI, −0.66 to −0.10; *p* = 0.01; *I*^2^ = 36.57%), and the parafovea region (1.0–3.0 mm from the fovea; SMD, −0.48; 95% CI, −0.71 to −0.25; *p* < 0.001; *I*^2^ = 41.55%) (Supplementary Figures 1–3). The *E*-values would be 2.18 and 2.47 for AD risk and OCTA measurements to nullify the above pooled SMDs. There was no significant difference in superficial microvascular density in other regions, including superior, inferior, nasal, and temporal.

### Deep Capillary Plexus VD

Vessel density of DCP was significantly decreased in the AD and MCI groups compared to controls (Figures 3, 4). Microvascular densities of DCP were significantly decreased in AD vs. controls (SMD, −1.19; 95% CI, −2.00 to −0.38; *p* < 0.001; *I*^2^ = 92.38%), and MCI vs. controls (SMD, −0.53; 95% CI, −0.85 to −0.22; *p* < 0.001; *I*^2^ = 59.68%). To nullify the above pooled SMD estimates of vessel density in DCP, an unmeasured confounder would need to be associated with a risk ratio of 5.35 and 2.62 for AD or MCI risk and OCTA measurements, respectively. The results of subsector and quadrantal analyses did not show significant differences in deep microvascular loss between AD and healthy controls, which may be due to the limited numbers of studies reporting the subsector DCP in subjects with AD or MCI.

### Radial Peripapillary Capillary (RPC) VD

Vessel density of RPC revealed no significant difference in the AD and MCI groups compared to controls (Figures 3, 4).

### Foveal Avascular Zone (FAZ) Area

**Figure 5:**
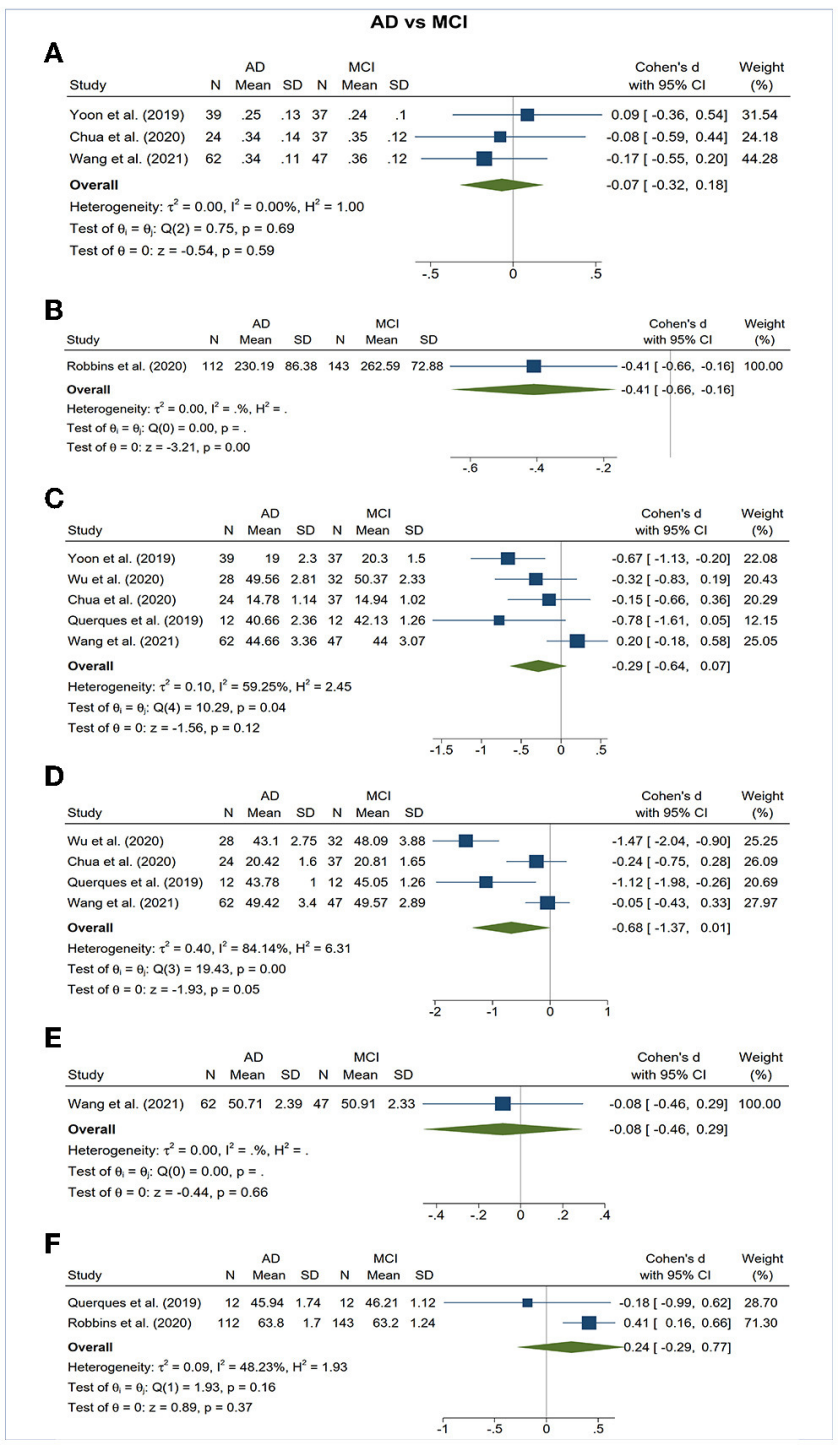
Forest plot of OCTA measurements between subjects with Alzheimer's Disease (AD) and mild cognitive impairment (MCI). The meta-analyses were conducted with a random-effects model. Horizontal bar indicates 95% confidence intervals (CI), and the size of the squares denotes the weight attributed to each article. The diamonds represent the standardized mean differences with the width showing the 95% CI. **(A)** Foveal avascular zone area. **(B)** Choroidal thickness. **(C)** Superficial capillary plexus vessel density. **(D)** Deep capillary plexus vessel density. **(E)** Peripapillary vessel density. **(F)** Choroidal perfusion density.

The area of the FAZ was significantly larger in AD subjects compared to controls (SMD, 0.54; 95% CI, 0.09 to 0.99; *p* = 0.02; *I*^2^ = 84.07%) (Figure 3). To nullify the pooled SMD estimates of FAZ, an unmeasured confounder would need to be associated with a risk ratio of 2.65 for AD risk and OCTA measurements. There was no statistical difference in the FAZ area between either AD subjects and MCI subjects or MCI subjects and controls (Figures 4, 5).

### Choroidal Thickness

There was no statistical difference in choroidal thickness between either AD subjects and MCI subjects compared to controls (Figures 3, 4).

### Meta-Regression

We performed meta-regression on studies comparing mean MMSE scores, mean age, gender ratio, OCT model, and macular scan size macular thickness between AD, MCI, and controls (Table 3). Our results showed significant associations between the type of OCTA model and the effect sizes of the foveal avascular zone between AD and controls (β = 0.923; *p* = 0.008; *r*^2^ = 53.72%). Furthermore, there were also significant associations between the macular scan size and the effect sizes of the area of foveal avascular zone between MCI and controls (β = 0.543, *p* = 0.030; *r*^2^ = 71.57%), the effect sizes of the SCP differences between AD and controls (β = 0.422, *p* = 0.008; *r*^2^ = 100%), and effect sizes of the DCP differences between AD and controls (β = −1.438, *p* = 0.031; *r*^2^ = 47.34%). All other factors had no significant impact (*p* > 0.05) on the effect sizes of the differences of VD in SCP and DCP or FAZ in AD, MCI groups.

**Table 3:** Results of meta-regression analysis: we performed random-effects meta-regression to assess the impact of study characteristics and potential confounders on the effect sizes of OCTA measurements, using SMD as the outcome variable.

	**Mean MMSE score differences**		**Age differences**		**Gender ratio differences**		**OCTA models (RTVue vs. Cirrus 5000)**		**Macular scan size (3x3 vs. 6x6)**	
	**β coefficient**	***p*** **-value**	**β coefficient**	***p*** **-value**	**β coefficient**	***p*** **-value**	**β coefficient**	***p*** **-value**	**β coefficient**	***p*** **-value**
**FAZ (AD vs. Controls)**										
Mean	−0.186	0.157	−0.168	**0.047**	1.157	0.545	0.923	**0.008**	0.673	0.139
**FAZ (MCI vs. Controls)**										
Mean	0.089	0.385	−0.056	0.554	−1.867	0.254	0.165	0.620	0.543	**0.030**
**SCP vessel density (AD vs. Controls)**										
Mean	0.093	0.129	0.032	0.635	−0.457	0.658	−0.069	0.774	0.422	**0.008**
**SCP vessel density (MCI vs. Controls)**										
Mean	−0.189	0.124	0.077	0.451	−0.730	0.750	−0.446	0.257	−0.078	0.860
**DCP vessel density (AD vs. Controls)**										
Mean	0.526	0.265	−0.026	0.937	−1.070	0.774	−0.336	0.730	−1.438	**0.031**
**DCP vessel density (MCI vs. Controls)**										
Mean	0.028	0.817	0.121	**0.050**	1.583	0.439	−0.419	0.152	−0.326	**0.325**

*p-values marked in bold indicate significance on the 95% confidence limit*.

*AD, Alzheimer's Disease; MCI, Mild cognitive impairment; OCTA, Optical coherence tomography angiography; MMSE, Mini–Mental State Examination; SCP, Superficial capillary plexus; DCP, Deep capillary plexus; FAZ, Foveal avascular zone*.

### Subgroup Analysis

Subgroup analyses were performed to compare the effect sizes between studies using RTVue XR Avanti and Cirrus 5000 Angioplex, and studies with a macular scan size of 3x3 and 6x6 mm. We observed that the differences of FAZ area between AD and controls were only statistically significant (*p* < 0.05) among studies using RTVue XR Avanti and a macular scan size of 6x6 mm. Likewise, the differences in FAZ area between MCI and controls were only statistically significant among studies with a macular scan size of 6x6 mm.

Regarding VD, the differences of SCP between MCI and controls was only statistically significant among studies with a macular scan size of 6x6 mm (Supplementary Figures 4, 5).

### Publication Bias and Risk of Bias

Egger's tests showed no publication bias in most of the analyses (*P* ≥ 0.05) (Table 4), except for the analyses of the FAZ area and DCP vessel density between AD and healthy controls. Most of the eligible studies were of low risk of bias (Table 5, Figure 2).

**Table 4:** Summary of differences of OCTA measurements and risk of publication bias using Egger's test.

	**Overall effect**		**Heterogeneity**		**Egger's test**	**Overall effect**		**Heterogeneity**		**Egger's test**	**Overall effect**		**Heterogeneity**		**Egger's test**
	**SMD**	***p*** **-value**	***I*** **^2^**	**Q (P)**		**SMD**	***p*** **-value**	***I*** **^2^**	**Q (P)**		**SMD**	***p*** **-value**	***I*** **^2^**	**Q (P)**	
	**AD vs. Controls**					**MCI vs. Controls**					**AD vs. MCI**				
FAZ	0.54 [0.09, 0.99]	0.02	84.1%	<0.001	0.0002	0.27 [−0.03, 0.57]	0.08	58.2%	0.04	0.848	−0.07 [−0.32, 0.18]	0.59	0.0%	0.69	0.651
Choroidal thickness	−0.58 [−1.26, 0.10]	0.09	88.4%	<0.001	0.436	-	-	-	-	-	-	-	-	-	-
SCP vessel density															
Mean	−0.48 [−0.70, −0.27]	<0.001	48.2%	0.04	0.227	−0.42 [−0.81, −0.03]	0.03	79.3%	<0.001	0.705	−0.29 [−0.64, 0.07]	0.12	59.3%	0.04	0.145
Fovea	−0.38 [−0.66, −0.10]	0.01	36.6%	0.19	0.025	−0.36 [−0.65, −0.07]	0.02	20.7%	0.26	0.573	0.04 [−0.61, 0.68]	0.91	75.5%	0.04	-
Parafovea	−0.48 [−0.71, −0.25]	<0.001	41.6%	0.10	0.217	−0.37 [−0.95, 0.21]	0.21	83.1%	<0.001	0.528	−0.31 [−0.82, 0.20]	0.23	74.4%	0.01	0.119
DCP vessel density	−1.19 [−2.00, −0.38]	<0.001	92.4%	<0.001	0.002	−0.53 [−0.85, −0.22]	<0.001	59.7%	0.02	0.968	−0.68 [−1.37, 0.01]	0.05	84.1%	<0.001	0.206
Peripapillary vessel density	−0.17 [−0.66, 0.31]	0.49	70.5%	0.04	0.221	−0.43 [−0.73, −0.14]	<0.001	0.0%	0.53	0.268	-	-	-	-	-
Choroidal perfusion density	−0.01 [−0.22, 0.20]	0.91	0.0%	0.73	0.719	−0.27 [−0.80, 0.26]	0.31	61.3%	0.11	–	0.24 [−0.29, 0.77]	0.37	48.2%	0.16	–

*SCP, Superficial capillary plexus; DCP, Deep capillary plexus; FAZ, Foveal avascular zone*.

**Table 5:** Risk of bias summary using the QUADAS-2 assessment.

**Study**	**Risk of bias**				**Applicability concerns**		
	**Patient selection**	**Index test**	**Reference standard**	**Flow and timing**	**Patient selection**	**Index test**	**Reference standard**
Bulut et al. (2018)	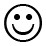	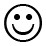	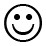	?	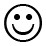	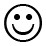	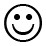
Chua et al. (2020)	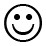	?	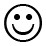	?	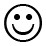	?	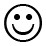
Criscuolo et al. (2020)	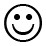	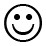	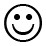	?	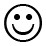	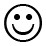	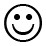
Haan et al. (2019)	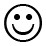	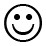	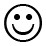	?	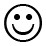	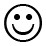	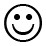
Jiang et al. (2018b)	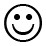	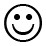	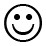	?	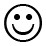	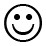	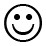
Lahme et al. (2018)	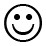	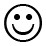	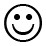	?	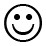	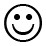	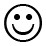
O'Bryhim et al. (2018)	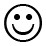	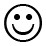	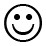	?	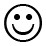	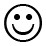	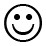
O'Bryhim et al. (2021)	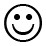	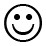	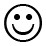	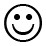	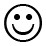	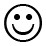	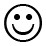
Querques et al. (2019)	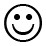	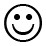	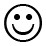	?	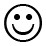	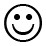	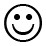
Robbins et al. (2021)	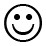	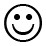	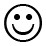	?	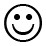	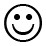	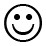
Salobrar-Garcia et al. (2020)	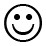	?	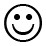	?	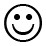	?	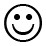
Shin et al. (2021)	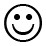	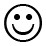	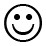	?	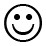	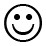	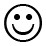
van de Kreeke et al. (2020)	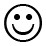	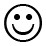	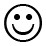	?	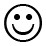	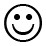	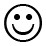
Wang et al. (2021)	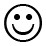	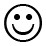	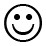	?	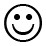	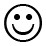	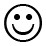
Wu et al. (2020)	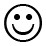	?	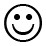	?	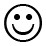	?	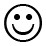
Yoon et al. (2019)	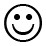	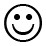	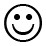	?	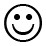	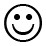	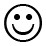
Zabel et al. (2019)	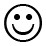	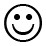	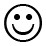	?	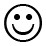	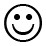	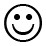
Zhang et al. (2019)	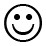	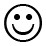	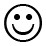	?	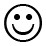	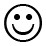	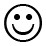

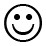
* = Low Risk*.

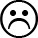
* = High Risk*.

*? = Unclear Risk*.

## Discussions

With vast improvement in imaging technique, the OCTA have enabled researchers to explore neurodegenerative changes of the retina as an extension of the CNS. To our knowledge, this is the first meta-analysis that analyzes all the available OCTA data to evaluate microvascular impairment in the eyes of AD and MCI patients. Our study demonstrated that AD and MCI patients exhibited enlarged FAZ areas, and decreased microvascular density of SCP and DCP. We also observed a choroidal thinning trend among patients with AD and MCI, although this relationship did not reach statistical significance. Our findings contributed additional knowledge about the differences of OCTA measurements between AD and MCI and found a trend of progressively lower VD in both SCP and DCP in AD relative to MCI. These observations suggest that VD and FAZ may be potential biomarkers in screening for AD and may be used to distinguish between MCI and AD.

Patients with AD in our study demonstrated enlarged FAZ size than healthy controls. In the central macula, the superficial and deep vascular plexuses form a capillary-free region named FAZ (Snodderly et al., 1992; Yu et al., 2005). The FAZ size reflects the status of the microcapillary perfusion in the macular area, and previous studies have documented a strong correlation with the severity of capillary non-perfusion (Bresnick et al., 1984; Zeffren et al., 1990; de Carlo et al., 2015; Spaide et al., 2018). Additionally, some studies have found a close relationship between aging and FAZ size (Yu et al., 2005; Gómez-Ulla et al., 2019). Bulut et al. was the first group to identify significantly enlarged FAZ in AD patients. Although there is controversy over the changes in FAZ, enlarged FAZ in AD is consistent with the hypothesis that the decreased angiogenesis due to the Aβ binding to VEGF and the accumulation of Aβ deposits in the internal vessel walls will lead to progressive capillary dropout (Berisha et al., 2007; Dorr et al., 2012).

Our analysis also showed that VD of the SCP is generally lower in both AD and MCI patients when compared to controls group, which supports most of the previous OCTA studies. Although the magnitude failed to reach a statistical significance in MCI, which may be partly due to a small number of eligible studies, the observed trend of retinal microvascular loss from MCI to AD may indicate paralleled retinal vascular impairment during disease progression. The SCP is supplied by the central retinal artery and located primarily in the retinal nerve fiber layer (RNFL) and ganglion cell layer (GCL; Campbell et al., 2017). Notably, a meta-analysis of 30 studies that included 1,257 AD, 305 MCI, and 1,460 control cases concluded significant macular changes, including GCL deficits in AD and MCI (Chan et al., 2019). Reduced number of retinal ganglion cells and axons has also been observed in post-mortem AD retina, possibly due to the impairment of microvascular structure responsible for the metabolic supply of these regions or vice versa (Hinton et al., 1986; Blanks et al., 1996). In agreement with this hypothesis, GCL abnormalities were also reported with other neurodegenerative diseases, including multiple sclerosis, neuromyelitis optica, and cerebral atrophy (Fisher et al., 2006; Frohman et al., 2008; Monteiro et al., 2012; Ong et al., 2015). Although the mechanism underlying reduced retinal VD remains elusive, one basic assumption is that Aβ protein deposits around vascular walls and disrupts the basement membrane of small vessels. Recent studies also identified substantial early pericyte loss together with significant Aβ deposition in retinal microvasculature, marking blood-brain barrier breakdown in AD (Halliday et al., 2016; Shi et al., 2020). Interestingly, in our subgroup analyses, we observed that the differences of VD in SCP were only statistically significant in the fovea and parafoveal region (Jiang et al., 2018b). Such density changes may be vulnerable to an earlier attack by pathophysiologic stress associated with retinal vascular damage.

Changes in the DCP seen in AD patients are further complementary to the findings in SCP and the structural thinning of the inner nuclear layer (INL; Santos et al., 2018; Jáñez-Escalada et al., 2019). The DCP is important for nurturing INL, which is principally composed of cell bodies involved in the vertical transmission of information from photoreceptors *via* their dendrites and axonal connections (Strettoi and Masland, 1995; Gupta et al., 2020). Noteworthily, Aβ deposits have also been detected in the INL in transgenic AD mouse models (Ning et al., 2008). Although the decreased VD of the DCP between AD and MCI, or even between MCI and control, did not reach the significant level, a strong correlation was seen in the forest plot and may likely present significance with more longitudinal studies in the future.

In addition to decreased VD in the retina, we observed choroidal thinning measured by OCTA in AD. This finding did not reach statistical significance due to the limited eligible studies, but it is consistent with previous studies using OCT for choroidal measurement (Gharbiya et al., 2014; Bayhan et al., 2015; Bulut et al., 2016; Cunha et al., 2017). The choroid is primarily a vascular structure providing nutrients and oxygen to the outer retina (Nickla and Wallman, 2010). Impairment of the oxygen flow from choroid to retina may cause degenerative change and neovascularization in diseases such as age-related macular degeneration (Pauleikhoff et al., 1999; Farazdaghi and Ebrahimi, 2019). Studies have reported the accumulation of Aβ protein within choroidal vessels in a normal aging mouse model and a transgenic AD mouse model (Miao et al., 2005; Cheung et al., 2014). Local Aβ deposits in choroid may induce inflammatory response and complements activation which inevitably lead to neurodegeneration and vasoregression of choroidal vasculature through a pathological cascade previously described in CNS in the patients with AD (Marchesi, 2011; Golzan et al., 2017; Salobrar-Garcia et al., 2020).

Subgroup analyses and meta-regression were performed to evaluate the heterogeneity across different studies and explore potential sources of bias. It is noteworthy that heterogeneity is to be expected in meta-analyses of diagnostic testing, and for this reason, a random-effects model was adopted in our study. Regarding the scan area of macula, previous studies suggested that retinal microvasculature is more vulnerable to an earlier attack in the para-macular area, and loss of macular microvasculature is more likely to originate from the parafoveal annular zone (Jiang et al., 2018b). Similarly, the results of our meta-regression showed that the scan area had a significant impact on the effect sizes regarding the differences of VD in SCP and DCP and the differences of FAZ. Macular scan size of 6x6 mm^2^ appeared to be more sensitive in detecting early change of microvasculature especially in DCP. Likewise, RTVue XR Avanti demonstrated a more consistent finding in FAZ changes between AD and controls, which is likely a reason for the heterogeneity observed in the meta-analysis. This may be explained by differences in the integrated projection artifact removal software between Optovue RTVue XR Avanti and Zeiss Cirrus 5000 Angioplex, and the split spectrum amplitude decorrelation angiography (SSADA) algorithm employed on the Optovue system. A prior study comparing these two OCTA systems found vessel discontinuity at the FAZ border in eyes imaged by the Zeiss but not by the Optovue (Chung et al., 2018). Similarly, a study compared four OCTA systems and found that the FAZ borders were best discernable on the Optovue followed by three other modules (Heidelberg, Topcon, and Zeiss; Munk et al., 2017). The SSADA algorithm employed on the Optovue uses a four-fold spectrum split, which may have enhanced the signal to noise ratio and provide a clean and continuous microvascular network inside the FAZ (Huang et al., 2016).

### Strengths and Limitations

The strengths of this meta-analysis lie in the comprehensive search and review, including the latest related studies, which helped us strengthen the findings of each study and obtain stronger evidence. Furthermore, this is to date the largest meta-analysis evaluating the role of OCTA in detecting early retinal microvasculature changes, including not only AD but MCI individuals. As recent studies suggest a critical window to early initiation of therapy, including MCI subjects may be essential to identify early pathologic biomarkers (Chalermpalanupap et al., 2013). Our study also provides a more precise estimate of the effect size and increases the generalizability of individual studies by performing stringent meta-regression and subgroup analyses. Even though heterogeneity remains among the eligible studies, we attempted to adjust for possible confounding bias in the meta-regression analysis to address the heterogeneity.

Our study has some limitations, and we have highlighted several knowledge gaps and priority needs for future research. First, there was a limited number of prospective studies included in our study. More prospective and longitudinal studies are required to provide comprehensive insights into the disease mechanism. Second, the number of studies may not be sufficient to achieve adequate statistical power in some sub-sector analyses, although the consistent trend of microvascular changes observed in AD and MCI subjects was still evident. Third, as previously discussed, pre-clinical AD and MCI subjects in different studies were not well-defined due to lack of disease-specific biomarkers and uniformed diagnostic criteria. Finally, parameters for image acquisition among different OCTA models should be documented in a standard format to compare these data across institutions reliably.

The retina provides a unique opportunity and correlation with AD. With the rise of OCTA, the potential biomarkers for early detection of AD are brighter than ever. Our findings in this meta-analysis further emphasize the role, contribution, and importance of the retinal microvasculature in the pathogenesis of AD, and may provide a roadmap for other neurodegenerative diseases.

## Conclusion

In summary, our study confirmed the retinal and choroidal microvasculature deficits from OCTA measurements in AD and MCI when compared to controls, highlighting the potential role of OCTA biomarkers in AD screening, diagnosis, and disease management. As the population ages rapidly and the prevalence of neurodegenerative diseases continues to rise, we anticipate that retinal biomarkers will play a major role in detecting and monitoring disease progression.

## Data Availability Statement

The original contributions presented in the study are included in the article/Supplementary Material, further inquiries can be directed to the corresponding author.

## Author Contributions

T-CY and C-TK conceived and designed the study, extracted the data, and wrote the manuscript. T-CY, C-TK, and Y-BC analyzed the data, verified the data, and revised the manuscript. All authors read and approved the final manuscript.

## Conflict of Interest

The authors declare that the research was conducted in the absence of any commercial or financial relationships that could be construed as a potential conflict of interest.

## Publisher's Note

All claims expressed in this article are solely those of the authors and do not necessarily represent those of their affiliated organizations, or those of the publisher, the editors and the reviewers. Any product that may be evaluated in this article, or claim that may be made by its manufacturer, is not guaranteed or endorsed by the publisher.

## Abbreviations

AD: Alzheimer's disease
MCI: Mild cognitive impairment
aMCI: Amnestic mild cognitive impairment
VD: vessel density
OR: Odds ratio
CI: Confidence interval
SMD: Standardized mean difference
CC: Case–control study
PS: Prospective study
MMSE: Mini-Mental State Examination
NIA-AA: National Institute on Aging and Alzheimer's Association
NINCDS-ADRDA: National Institute of Neurological, Communicative Disorders and Stroke-Alzheimer Disease and Related Disorders Association
CDR: Clinical Dementia Rating
TICS-M: The modified Telephone Interview for Cognitive Status
DSM-IV: Diagnostic and Statistical Manual of Mental Disorders, fourth edition
OCTA: Optical Coherence Tomography Angiography
Eye
S: OCTA measurements of either single eye were selected
P: OCTA measurements of both eyes were considered
SCP: Superficial capillary plexus
DCP: Deep capillary plexus
FAZ: Foveal avascular zone.
