# Supplementary material for: Retinal Microvascular Changes in Mild Cognitive Impairment and Alzheimer's Disease: A Systematic Review, Meta-Analysis, and Meta-Regression
Source: Front Aging Neurosci. 2022 Apr 28;14:860759. doi: 10.3389/fnagi.2022.860759 (PMC9096234; doi:10.3389/fnagi.2022.860759)
Supplement: Supplementary file 1 [file Data_Sheet_1.pdf]

SUPPLEMENTAL FIGURES

AD vs Control

A. Superficial Capillary Plexus Vessel Density: Fovea (<1.0 mm to the Fovea)

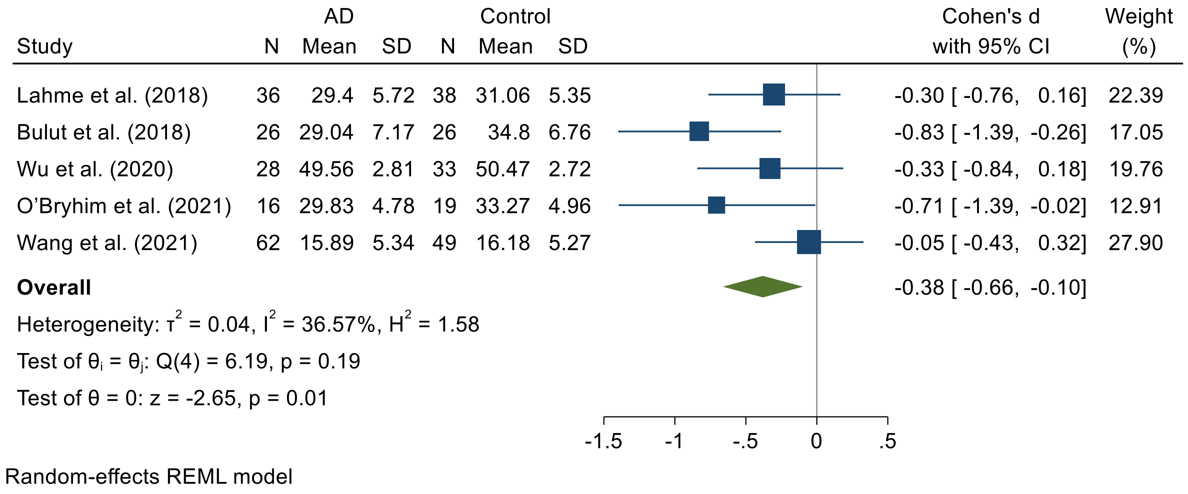

B. Superficial Capillary Plexus Vessel Density: Parafovea region (1.0–3.0 mm to the Fovea)

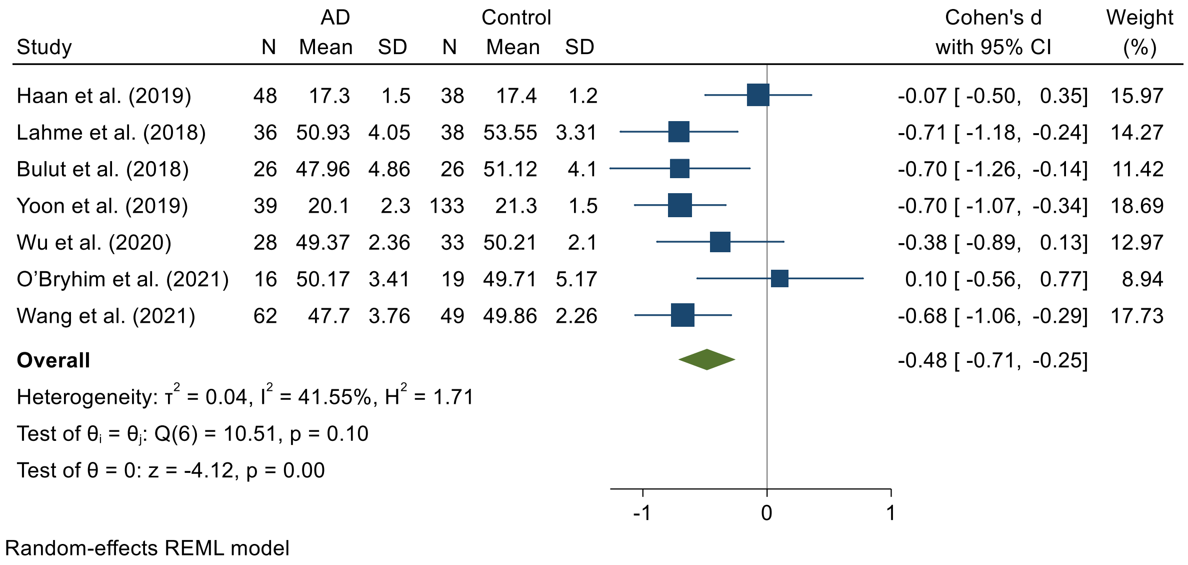

Figure S1. Difference in the superficial capillary plexus vessel density between subjects with Alzheimer's Disease (AD) and healthy controls

The meta-analyses were conducted with a random-effects model. Horizontal bar indicates 95% confidence intervals (CI), and the size of the squares denotes the weight attributed to each article. The diamonds represent the standardized mean differences with the width showing the 95% CI.

MCI vs Control

A. Superficial Capillary Plexus Vessel Density: Fovea (<1.0 mm to the Fovea)

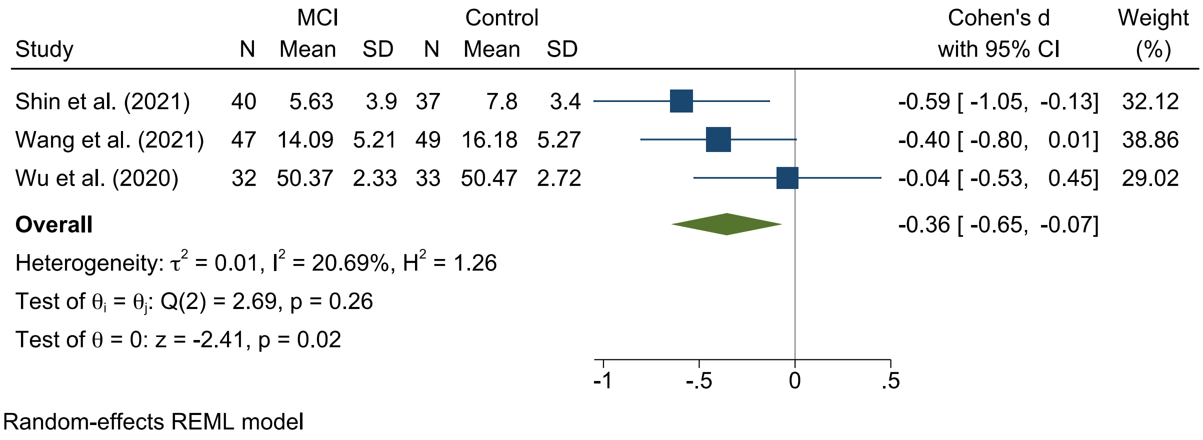

B. Superficial Capillary Plexus Vessel Density: Parafovea region (1.0–3.0 mm to the Fovea)

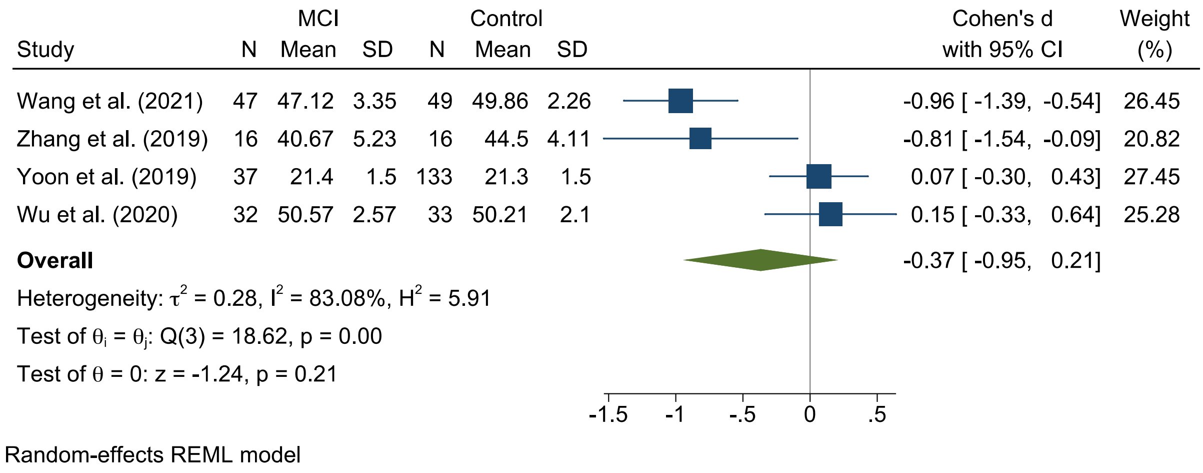

Figure S2. Difference in the superficial capillary plexus vessel density between subjects with mild cognitive impairment (MCI) and healthy controls

The meta-analyses were conducted with a random-effects model. Horizontal bar indicates 95% confidence intervals (CI), and the size of the squares denotes the weight attributed to each article. The diamonds represent the standardized mean differences with the width showing the 95% CI.

AD vs MCI

A. Superficial Capillary Plexus Vessel Density: Fovea (<1.0 mm to the Fovea)

Study	AD N	AD Mean	AD SD	MCI N	MCI Mean	MCI SD	Cohen's d with 95% CI	Weight (%)
Wu et al. (2020)	28	49.56	2.81	32	50.37	2.33	-0.32 [-0.83, 0.19]	46.54
Wang et al. (2021)	62	15.89	5.34	47	14.09	5.21	0.34 [-0.04, 0.72]	53.46
**Overall**							0.04 [-0.61, 0.68]	

Heterogeneity: τ^2 = 0.16, I^2 = 75.46%, H^2 = 4.08

Test of $\theta_i = \theta_j$: Q(1) = 4.08, p = 0.04

Test of θ = 0: z = 0.11, p = 0.91

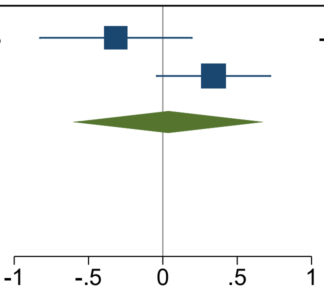

Random-effects REML model

B. Superficial Capillary Plexus Vessel Density: Parafovea region (1.0–3.0 mm to the Fovea)

Study	AD N	AD Mean	AD SD	MCI N	MCI Mean	MCI SD	Cohen's d with 95% CI	Weight (%)
Yoon et al. (2019)	39	20.1	2.3	37	21.4	1.5	-0.67 [-1.13, -0.20]	32.95
Wu et al. (2020)	28	49.37	2.36	32	50.57	2.57	-0.48 [-1.00, 0.03]	30.97
Wang et al. (2021)	62	47.7	3.76	47	47.12	3.35	0.16 [-0.22, 0.54]	36.08
**Overall**							-0.31 [-0.82, 0.20]	

Heterogeneity: τ^2 = 0.15, I^2 = 74.42%, H^2 = 3.91

Test of $\theta_i = \theta_j$: Q(2) = 8.43, p = 0.01

Test of θ = 0: z = -1.19, p = 0.23

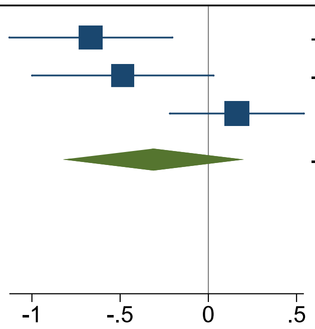

Random-effects REML model

Figure S3. Difference in the superficial capillary plexus vessel density between subjects with Alzheimer's Disease (AD) and mild cognitive impairment (MCI)

The meta-analyses were conducted with a random-effects model. Horizontal bar indicates 95% confidence intervals (CI), and the size of the squares denotes the weight attributed to each article. The diamonds represent the standardized mean differences with the width showing the 95% CI.

Foveal Avascular Zone Area: AD vs Control

Study	AD N	AD Mean	AD SD	Control N	Control Mean	Control SD	Cohen's d with 95% CI	Weight (%)
**Cirrus 5000 Angioplex**								
Haan et al. (2019)	48	.24	.06	38	.26	.08	-0.29 [-0.72, 0.14]	13.50
Yoon et al. (2019)	39	.25	.13	133	.25	.11	0.00 [-0.36, 0.36]	14.02
Chua et al. (2020)	24	.34	.14	29	.31	.12	0.23 [-0.31, 0.77]	12.57
Heterogeneity: $\tau^2 = 0.00$, $I^2 = 0.80\%$, $H^2 = 1.01$							-0.05 [-0.29, 0.20]	
Test of $\theta_i = \theta_j$: Q(2) = 2.30, p = 0.32								
**RTVue XR Avanti**								
Bulut et al. (2018)	26	.47	.18	26	.33	.08	1.01 [0.43, 1.58]	12.28
Zabel et al. (2019)	27	.32	.09	27	.21	.07	1.36 [0.77, 1.96]	12.14
O'Bryhim et al. (2021)	16	.368	.077	19	.272	.083	1.20 [0.47, 1.92]	11.02
O'Bryhim et al. (2018)	14	.364	.095	16	.275	.06	1.14 [0.36, 1.91]	10.58
Wang et al. (2021)	62	.34	.11	49	.33	.12	0.09 [-0.29, 0.46]	13.89
Heterogeneity: $\tau^2 = 0.24$, $I^2 = 73.34\%$, $H^2 = 3.75$							0.91 [0.41, 1.42]	
Test of $\theta_i = \theta_j$: Q(4) = 19.21, p = 0.00								
**Overall**							0.54 [0.09, 0.99]	
Heterogeneity: $\tau^2 = 0.34$, $I^2 = 84.07\%$, $H^2 = 6.28$								
Test of $\theta_i = \theta_j$: Q(7) = 39.73, p = 0.00								
Test of group differences: $Q_b(1) = 11.15$, p = 0.00								

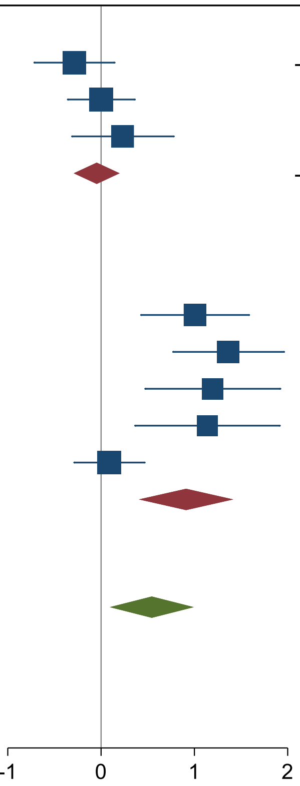

Random-effects REML model

Foveal Avascular Zone Area: MCI vs Control

Study	MCI N	MCI Mean	MCI SD	Control N	Control Mean	Control SD	Cohen's d with 95% CI	Weight (%)
**Cirrus 5000 Angioplex**								
Yoon et al. (2019)	37	.24	.1	133	.25	.11	-0.09 [-0.46, 0.27]	20.51
Chua et al. (2020)	37	.35	.12	29	.31	.12	0.33 [-0.16, 0.82]	16.49
Shin et al. (2021)	40	.31	.11	37	.27	.09	0.40 [-0.05, 0.85]	17.65
Heterogeneity: $\tau^2 = 0.04$, $I^2 = 42.28\%$, $H^2 = 1.73$							0.18 [-0.14, 0.51]	
Test of $\theta_i = \theta_j$: Q(2) = 3.38, p = 0.18								
**RTVue XR Avanti**								
Zhang et al. (2019)	16	.247	.092	16	.271	.135	-0.21 [-0.90, 0.49]	11.41
Criscuolo et al. (2020)	27	.28	.12	29	.19	.06	0.96 [0.41, 1.51]	14.69
Wang et al. (2021)	47	.36	.12	49	.33	.12	0.25 [-0.15, 0.65]	19.25
Heterogeneity: $\tau^2 = 0.23$, $I^2 = 75.41\%$, $H^2 = 4.07$							0.35 [-0.28, 0.98]	
Test of $\theta_i = \theta_j$: Q(2) = 7.36, p = 0.03								
**Overall**							0.27 [-0.03, 0.57]	
Heterogeneity: $\tau^2 = 0.08$, $I^2 = 58.20\%$, $H^2 = 2.39$								
Test of $\theta_i = \theta_j$: Q(5) = 11.89, p = 0.04								
Test of group differences: $Q_b(1) = 0.22$, p = 0.64								

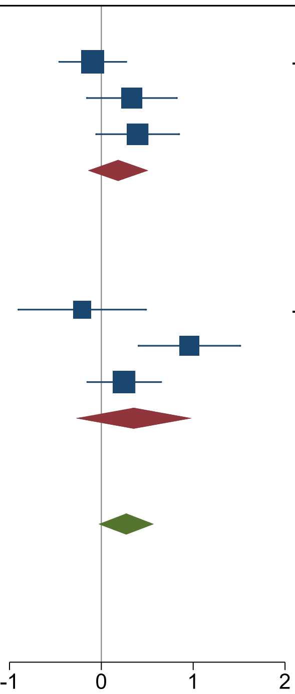

Random-effects REML model

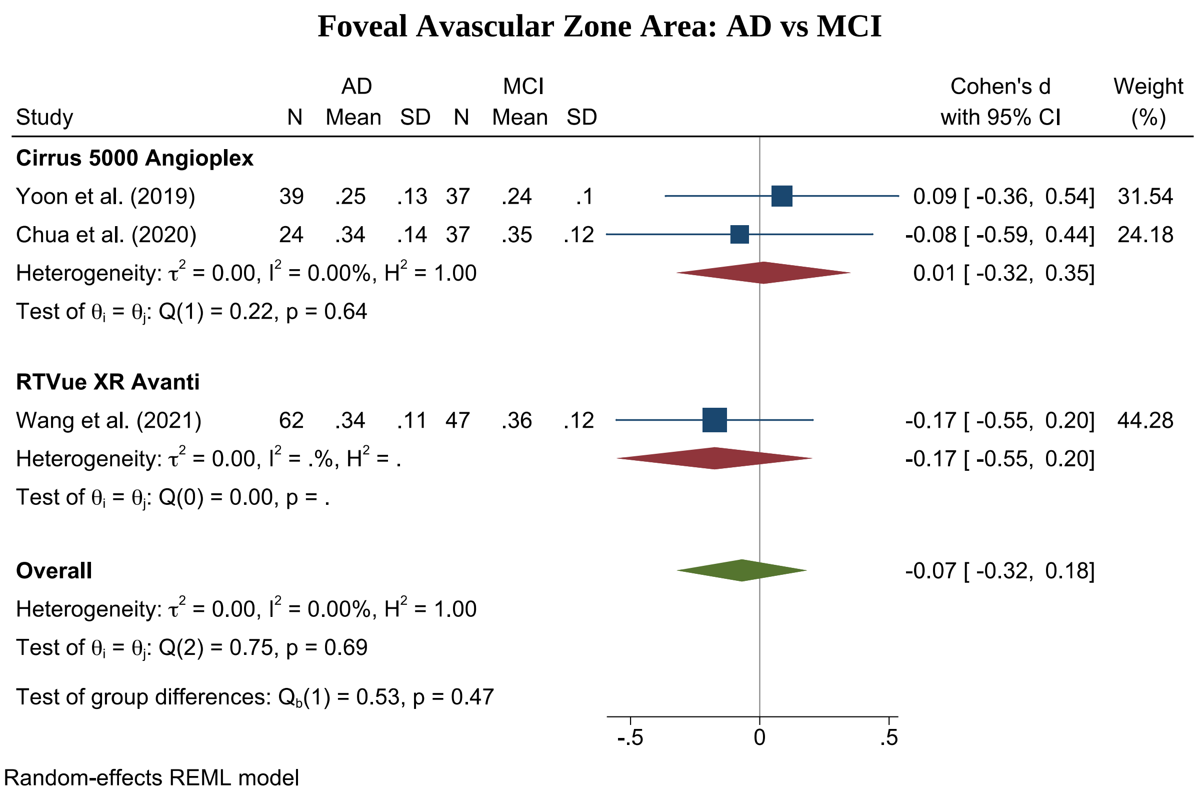

Figure S4. Subgroup analyses comparing studies using Cirrus 5000 Angioplex and RTVue XR Avanti

The meta-analyses were conducted with a random-effects model. Horizontal bar indicates 95% confidence intervals (CI), and the size of the squares denotes the weight attributed to each article. The diamonds represent the standardized mean differences with the width showing the 95% CI. Abbreviation: AD = Alzheimer's Disease; MCI = Mild Cognitive Impairment

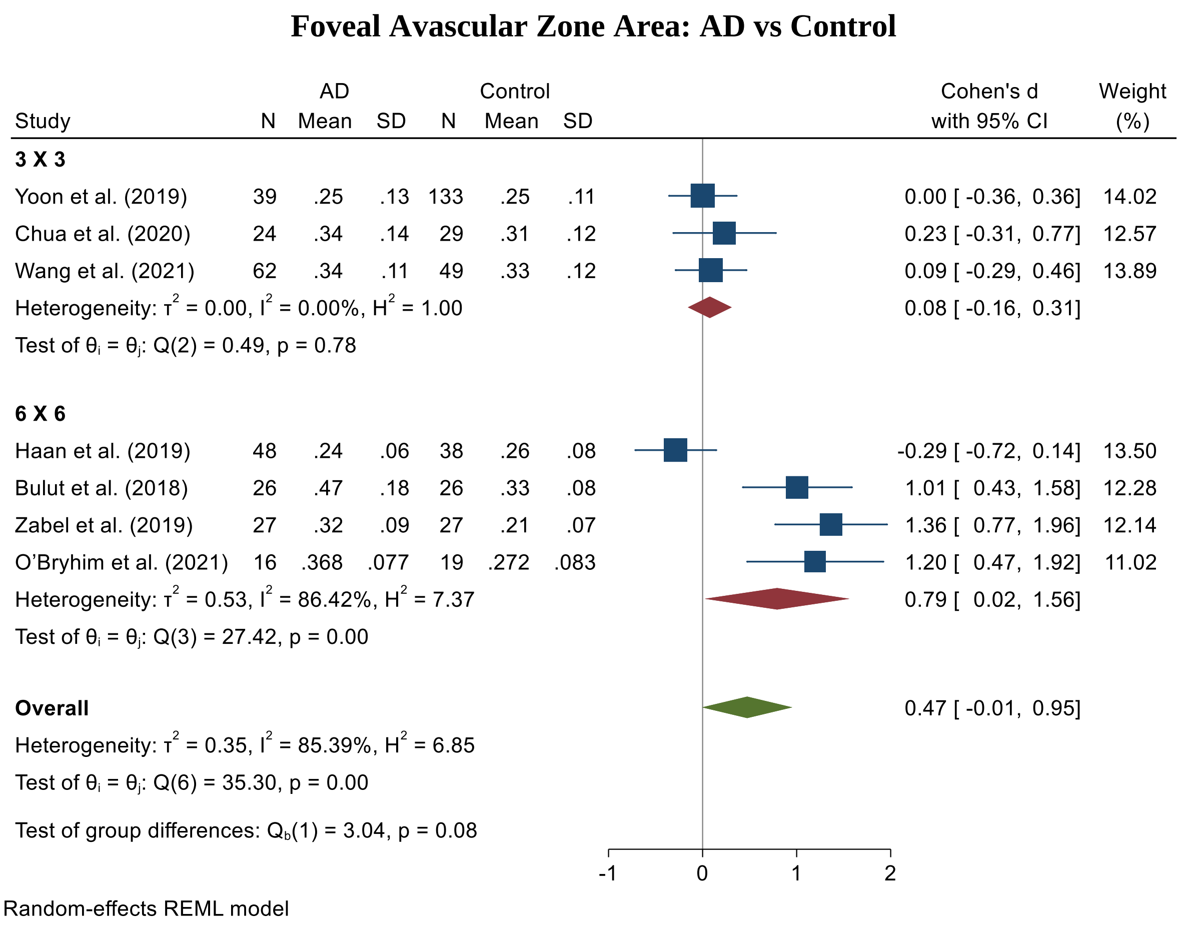
Foveal Avascular Zone Area: AD vs Control
AD
Control
Cohen's d with 95% CI
Weight (%)
Study N Mean SD N Mean SD
3 X 3
Yoon et al. (2019) 39 .25 .13 133 .25 .11 0.00 [-0.36, 0.36] 14.02
Chua et al. (2020) 24 .34 .14 29 .31 .12 0.23 [-0.31, 0.77] 12.57
Wang et al. (2021) 62 .34 .11 49 .33 .12 0.09 [-0.29, 0.46] 13.89
Heterogeneity: τ^2 = 0.00, I^2 = 0.00%, H^2 = 1.00 0.08 [-0.16, 0.31]
Test of $\theta_i = \theta_j$: Q(2) = 0.49, p = 0.78
6 X 6
Haan et al. (2019) 48 .24 .06 38 .26 .08 -0.29 [-0.72, 0.14] 13.50
Bulut et al. (2018) 26 .47 .18 26 .33 .08 1.01 [0.43, 1.58] 12.28
Zabel et al. (2019) 27 .32 .09 27 .21 .07 1.36 [0.77, 1.96] 12.14
O'Bryhim et al. (2021) 16 .368 .077 19 .272 .083 1.20 [0.47, 1.92] 11.02
Heterogeneity: τ^2 = 0.53, I^2 = 86.42%, H^2 = 7.37 0.79 [0.02, 1.56]
Test of $\theta_i = \theta_j$: Q(3) = 27.42, p = 0.00
Overall 0.47 [-0.01, 0.95]
Heterogeneity: τ^2 = 0.35, I^2 = 85.39%, H^2 = 6.85
Test of $\theta_i = \theta_j$: Q(6) = 35.30, p = 0.00
Test of group differences: $Q_b(1)$ = 3.04, p = 0.08
-1 0 1 2
Random-effects REML model

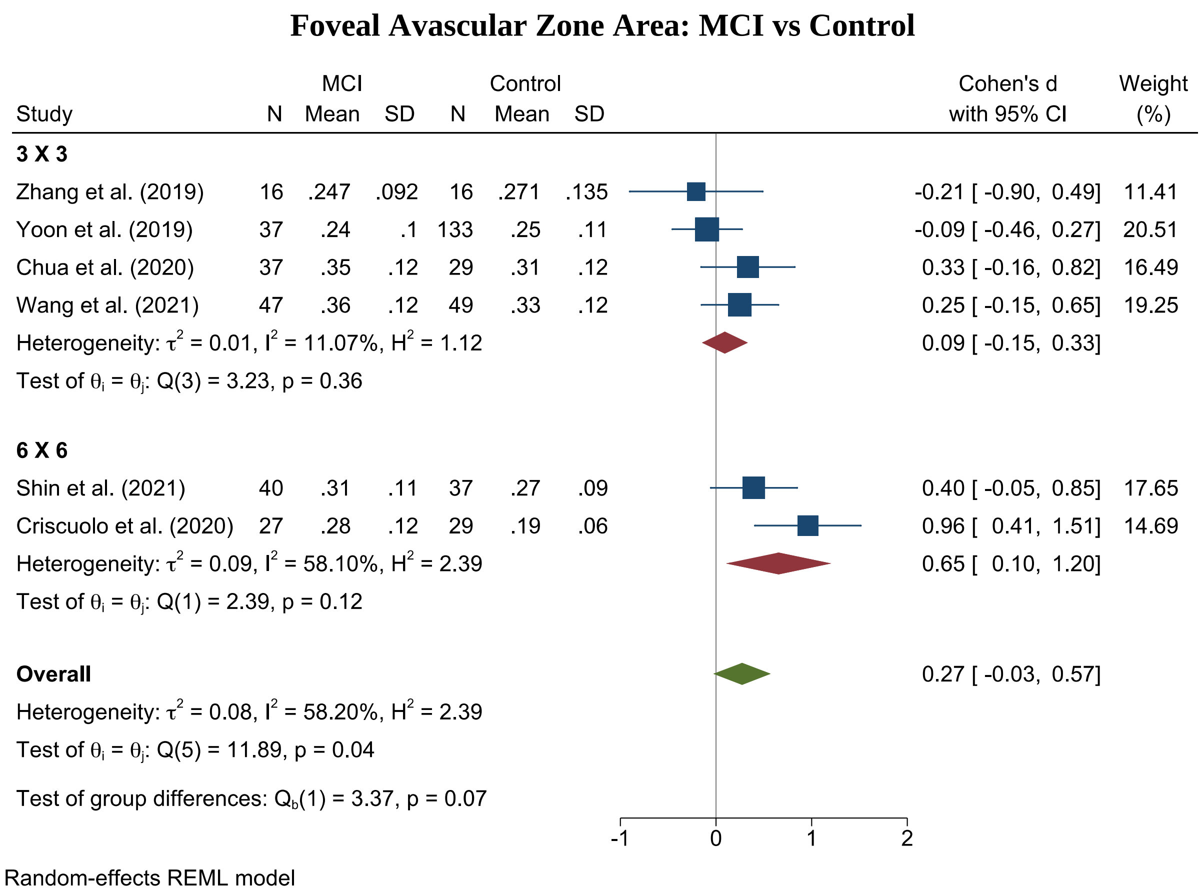
Foveal Avascular Zone Area: MCI vs Control
MCI
Control
Cohen's d with 95% CI
Weight (%)
Study N Mean SD N Mean SD
3 X 3
Zhang et al. (2019) 16 .247 .092 16 .271 .135 -0.21 [-0.90, 0.49] 11.41
Yoon et al. (2019) 37 .24 .1 133 .25 .11 -0.09 [-0.46, 0.27] 20.51
Chua et al. (2020) 37 .35 .12 29 .31 .12 0.33 [-0.16, 0.82] 16.49
Wang et al. (2021) 47 .36 .12 49 .33 .12 0.25 [-0.15, 0.65] 19.25
Heterogeneity: τ^2 = 0.01, I^2 = 11.07%, H^2 = 1.12 0.09 [-0.15, 0.33]
Test of $\theta_i = \theta_j$: Q(3) = 3.23, p = 0.36
6 X 6
Shin et al. (2021) 40 .31 .11 37 .27 .09 0.40 [-0.05, 0.85] 17.65
Criscuolo et al. (2020) 27 .28 .12 29 .19 .06 0.96 [0.41, 1.51] 14.69
Heterogeneity: τ^2 = 0.09, I^2 = 58.10%, H^2 = 2.39 0.65 [0.10, 1.20]
Test of $\theta_i = \theta_j$: Q(1) = 2.39, p = 0.12
Overall 0.27 [-0.03, 0.57]
Heterogeneity: τ^2 = 0.08, I^2 = 58.20%, H^2 = 2.39
Test of $\theta_i = \theta_j$: Q(5) = 11.89, p = 0.04
Test of group differences: $Q_b(1)$ = 3.37, p = 0.07
-1 0 1 2
Random-effects REML model

Superficial Capillary Plexus Vessel Density: AD vs Control

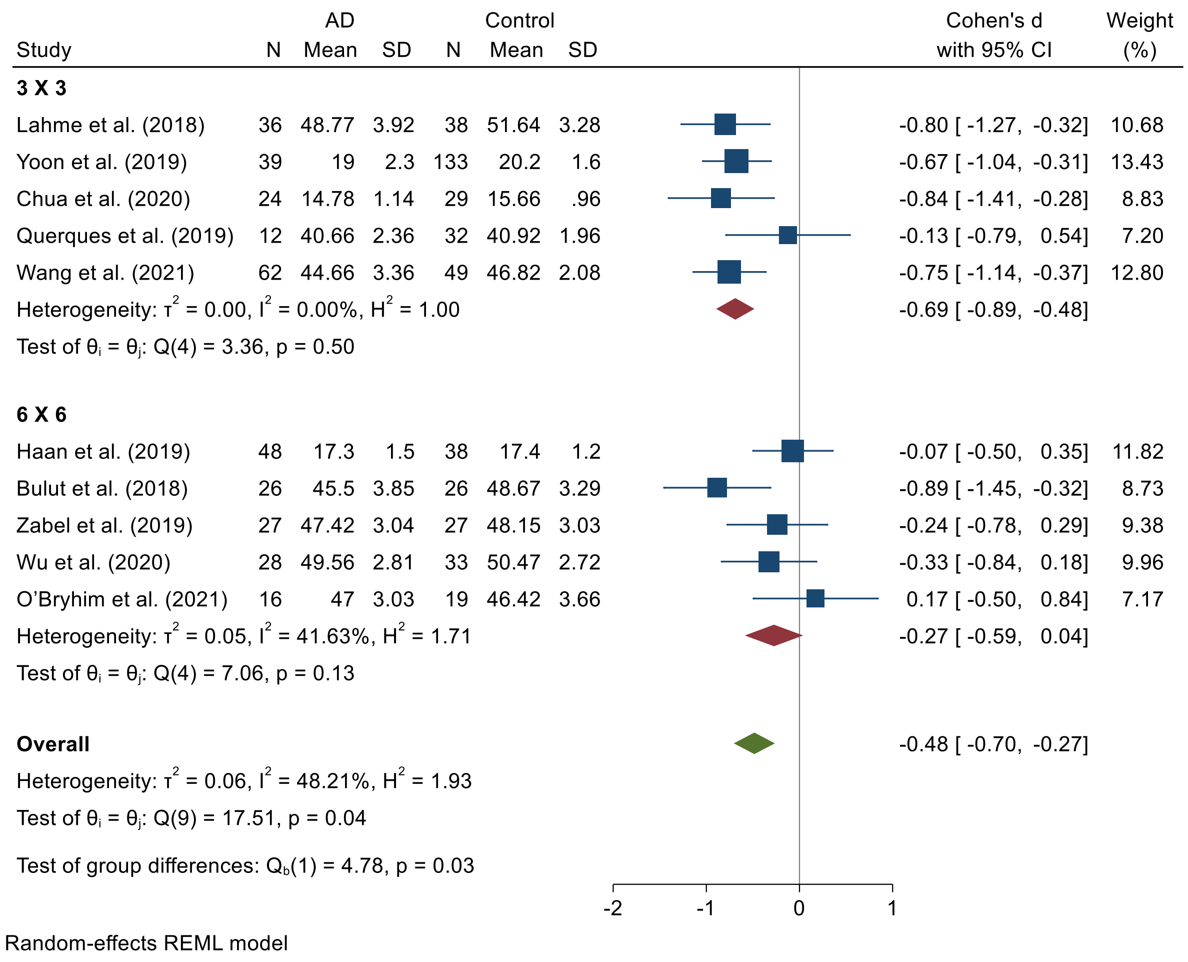

Superficial Capillary Plexus Vessel Density: MCI vs Control

Study	MCI N	MCI Mean	MCI SD	Control N	Control Mean	Control SD	Cohen's d with 95% CI	Weight (%)
**3 X 3**								
Zhang et al. (2019)	16	40.67	5.23	16	44.5	4.11	-0.81 [-1.54, -0.09]	10.39
Yoon et al. (2019)	37	20.3	1.5	133	20.2	1.6	0.06 [-0.30, 0.43]	14.14
Chua et al. (2020)	37	14.94	1.02	29	15.66	.96	-0.72 [-1.23, -0.22]	12.73
Querques et al. (2019)	12	42.13	1.26	32	40.92	1.96	0.67 [-0.01, 1.35]	10.83
Wang et al. (2021)	47	44	3.07	49	46.82	2.08	-1.08 [-1.51, -0.65]	13.51
Heterogeneity: $\tau^2 = 0.42$, $I^2 = 86.55\%$, $H^2 = 7.44$							-0.39 [-1.01, 0.23]	
Test of $\theta_i = \theta_j$: Q(4) = 28.34, p = 0.00								
**6 X 6**								
Wu et al. (2020)	32	50.37	2.33	33	50.47	2.72	-0.04 [-0.53, 0.45]	12.89
Shin et al. (2021)	40	14	3.9	37	16.3	2.5	-0.70 [-1.16, -0.24]	13.17
Criscuolo et al. (2020)	27	44.92	5.04	29	48.12	4.53	-0.67 [-1.21, -0.13]	12.33
Heterogeneity: $\tau^2 = 0.08$, $I^2 = 55.06\%$, $H^2 = 2.23$							-0.47 [-0.89, -0.04]	
Test of $\theta_i = \theta_j$: Q(2) = 4.46, p = 0.11								
**Overall**							-0.42 [-0.81, -0.03]	
Heterogeneity: $\tau^2 = 0.24$, $I^2 = 79.29\%$, $H^2 = 4.83$								
Test of $\theta_i = \theta_j$: Q(7) = 32.94, p = 0.00								
Test of group differences: $Q_b(1) = 0.04$, p = 0.84								

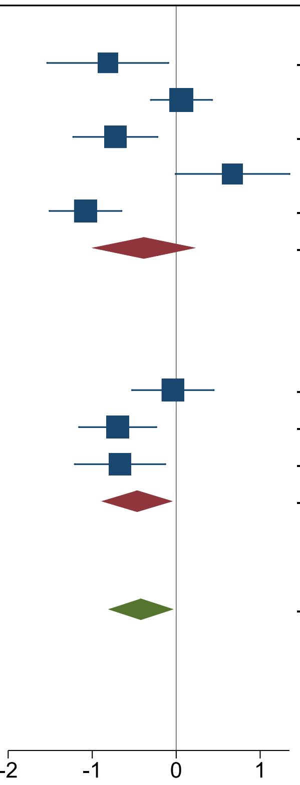

Random-effects REML model

Superficial Capillary Plexus Vessel Density: AD vs MCI

Study	AD N	AD Mean	AD SD	MCI N	MCI Mean	MCI SD	Cohen's d with 95% CI	Weight (%)
**3 X 3**								
Yoon et al. (2019)	39	19	2.3	37	20.3	1.5	-0.67 [-1.13, -0.20]	22.08
Chua et al. (2020)	24	14.78	1.14	37	14.94	1.02	-0.15 [-0.66, 0.36]	20.29
Querques et al. (2019)	12	40.66	2.36	12	42.13	1.26	-0.78 [-1.61, 0.05]	12.15
Wang et al. (2021)	62	44.66	3.36	47	44	3.07	0.20 [-0.18, 0.58]	25.05
Heterogeneity: $\tau^2 = 0.15$, $I^2 = 68.57\%$, $H^2 = 3.18$							-0.29 [-0.75, 0.17]	
Test of $\theta_i = \theta_j$: Q(3) = 10.14, p = 0.02								
**6 X 6**								
Wu et al. (2020)	28	49.56	2.81	32	50.37	2.33	-0.32 [-0.83, 0.19]	20.43
Heterogeneity: $\tau^2 = 0.00$, $I^2 = .\%$, $H^2 = .$							-0.32 [-0.83, 0.19]	
Test of $\theta_i = \theta_j$: Q(0) = 0.00, p = .								
**Overall**							-0.29 [-0.64, 0.07]	
Heterogeneity: $\tau^2 = 0.10$, $I^2 = 59.25\%$, $H^2 = 2.45$								
Test of $\theta_i = \theta_j$: Q(4) = 10.29, p = 0.04								
Test of group differences: $Q_b(1) = 0.01$, p = 0.94								

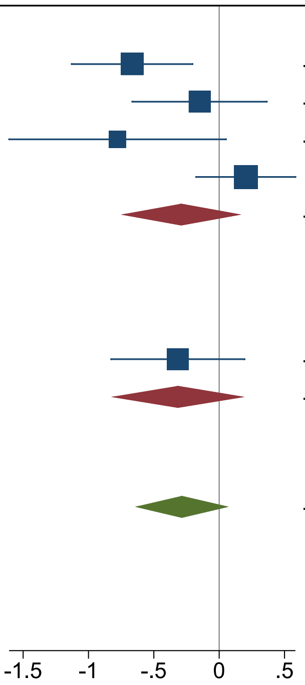

Random-effects REML model

Deep Capillary Plexus Vessel Density: AD vs Control

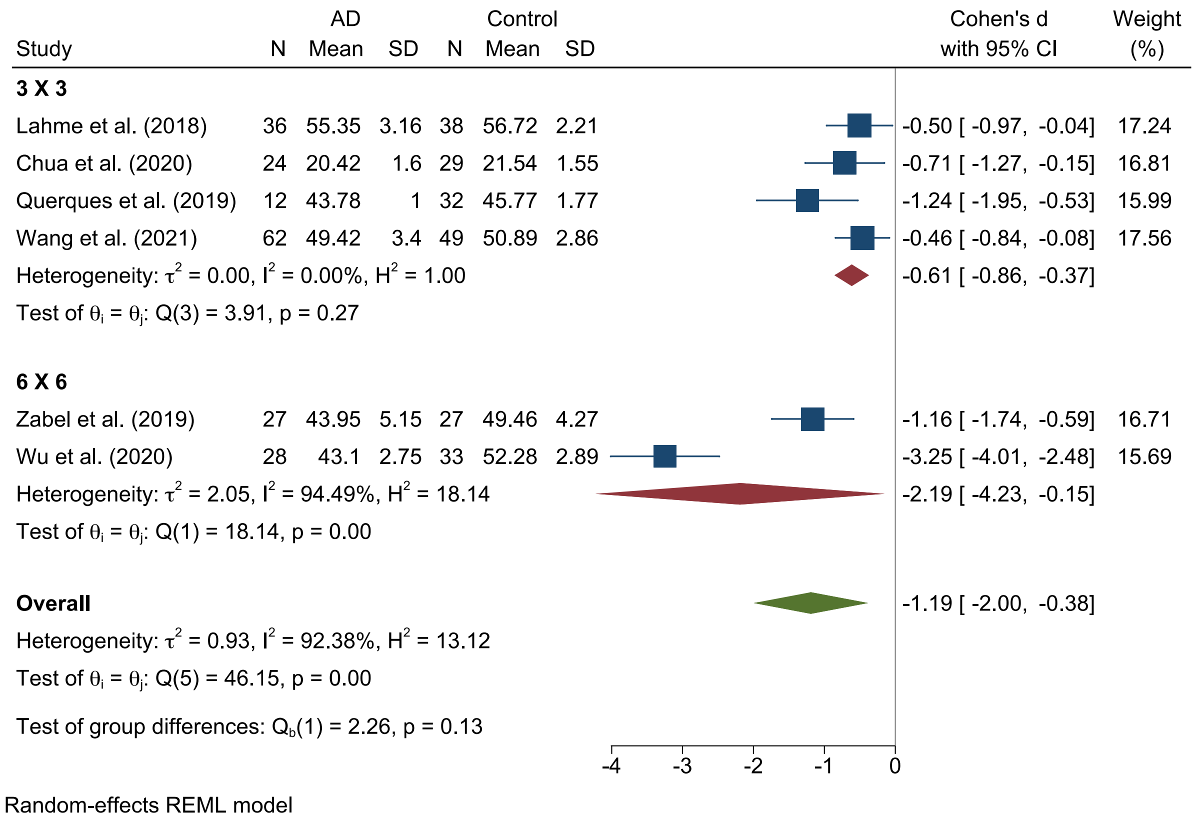

Deep Capillary Plexus Vessel Density: MCI vs Control

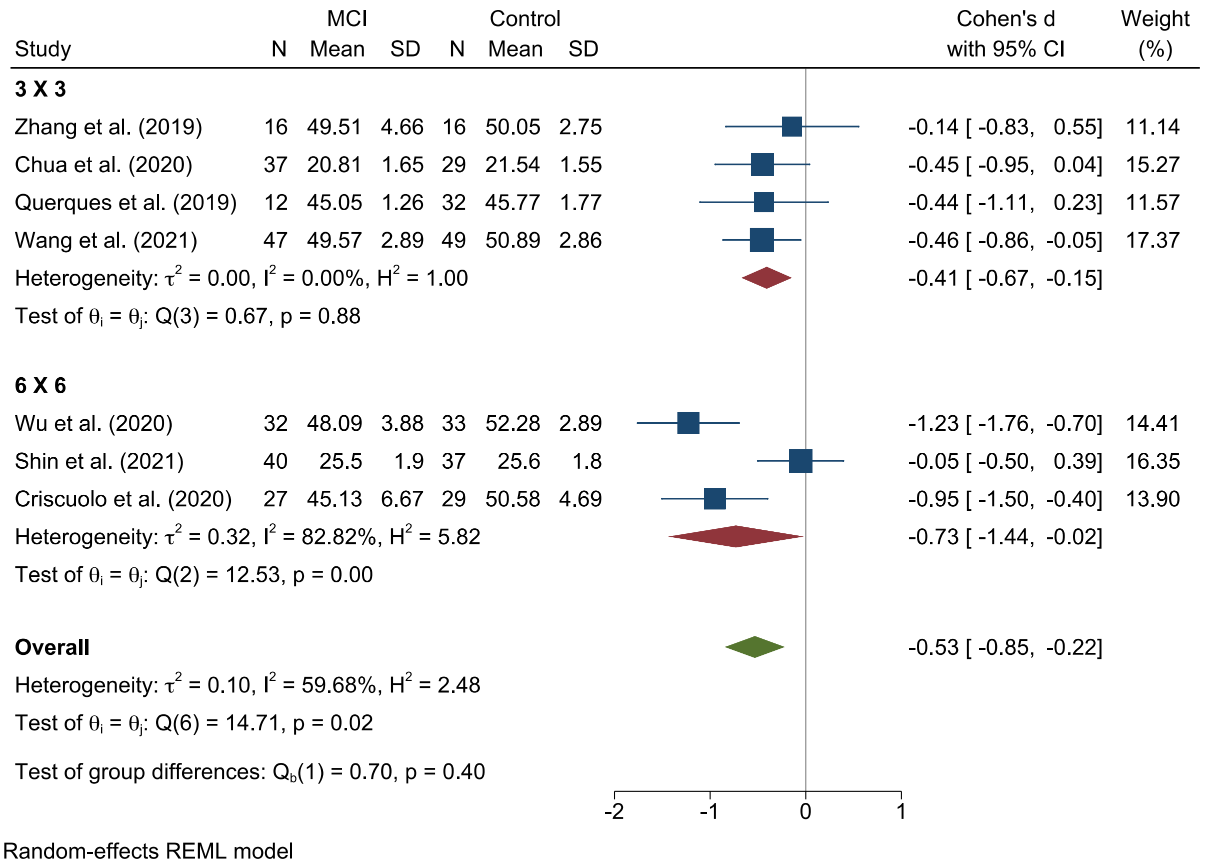

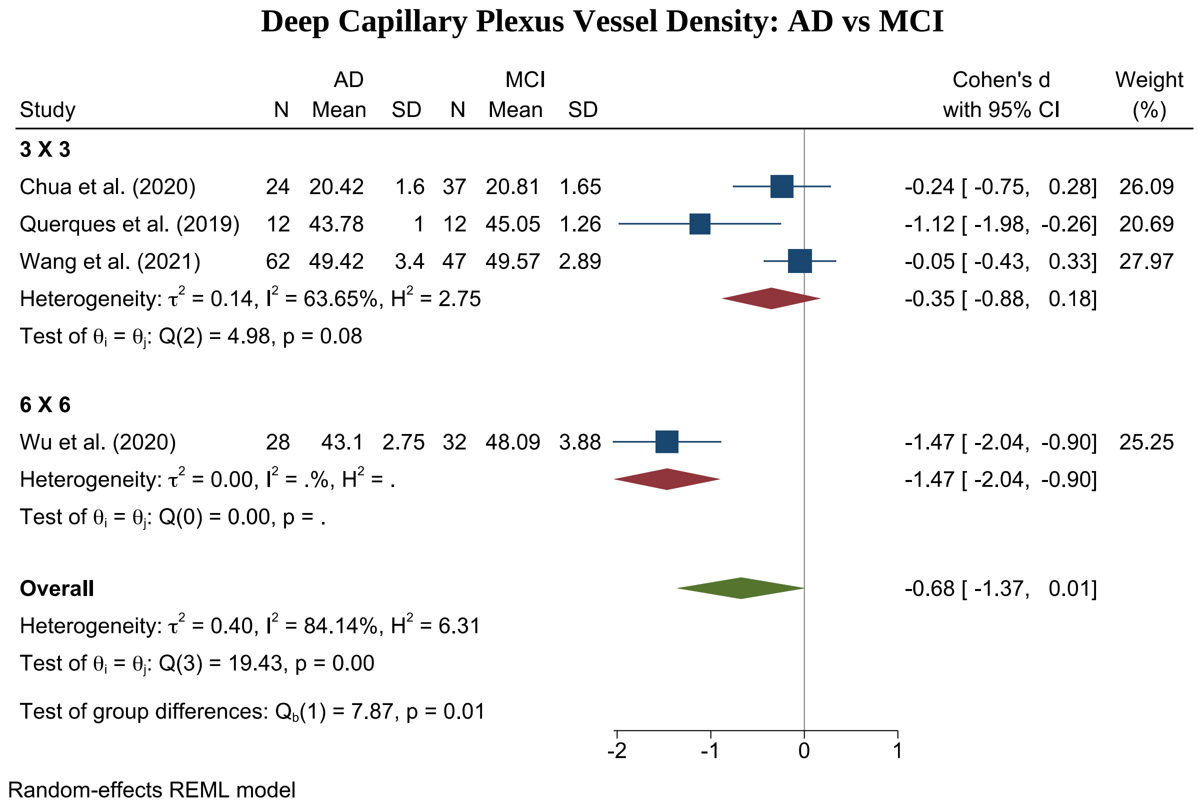

Figure S5. Subgroup analyses comparing studies with macular scan size of 3x3 mm and 6x6 mm

The meta-analyses were conducted with a random-effects model. Horizontal bar indicates 95% confidence intervals (CI), and the size of the squares denotes the weight attributed to each article. The diamonds represent the standardized mean differences with the width showing the 95% CI. Abbreviation: AD = Alzheimer's Disease; MCI = Mild Cognitive Impairment
